# Likes, comments, and shares of marine organism imagery on Facebook

**DOI:** 10.7717/peerj.6795

**Published:** 2019-04-24

**Authors:** Craig R. McClain

**Affiliations:** Louisiana Universities Marine Consortium, Chauvin, LA, United States of America

**Keywords:** Facebook, Virality, Science communication, Social media

## Abstract

Several calls to action urge scientists and science communicators to engage more with online communities. While these calls have been answered by a high percentage of scientists and science communicators online, it often remains unclear what are the best models for effective communication. Best practices and methods for online science communication can benefit from experimental and quantitative research addressing how and when users engage with online content. This study addresses with quantitative and predictive models a key question for the popular, but often-ignored in science communication, social media platform Facebook. Specifically, this study examines the impact of imagery through quantification of likes, comments, and shares on Facebook posts. Here, I show that a basic quantitative model can be useful in predicting response to marine organism imagery on Facebook. The results of this online experiment suggest image type, novelty, and aesthetics impact the number of likes, shares, and comments on a post. In addition, the likes, shares, and comments on images did not follow traditional definitions of “charismatic megafauna”, with cephalopods and bony fishes receiving more interactions than cartilaginous fishes and marine mammals. Length and quality of caption did not significantly impact likes, comments, or shares. This study provides one of the first quantitative analysis of virality of scientific images via social media. The results challenge previously held conceptions of social media scientific outreach including increasing emphasis on imagery selection and curation, notions of which taxa the public connect with, and role of captions for imagery.

## Introduction

Online environments play a fundamental and core role in social connections and dissemination of information. Many social media users, nearly 67%, get at least some news through social media, a percentage that rises to 75% percent among minorities (Shearer & Gottfried, 2017). These trends apply to other digital spaces, like blogs (Jarreau & Porter, 2018; Saunders et al., 2017). However, while adults interested in science prefer to get scientific information from the internet rather than from more traditional media (Takahashi & Tandoc, 2016), only one in six Americans actively seek out or frequently consume any science information and those mainly go through traditional media outlets, not science-focused channels (Funk, Gottfried & Mitchell, 2017). Moreover:

“Social media, while prominent as a general news source, appear to play a modest role in informing Americans about science. Most social media users see science-related posts on these platforms, though only a quarter (twenty-five percent) see “a lot” or “some” science posts; and a third (thirty-three percent) consider this an important way they get science news” (Funk, Gottfried & Mitchell, 2017).

Within this context, the necessity for scientists, communicators, and educators to engage with the social medial users about science has been the subject of an increasing number of calls to action from both within and outside the field of science communication (Ashlin & Ladle, 2006; Bik et al., 2015; Bik & Goldstein, 2013; Friedman, 2008; Ranganathan, 2013; Reddy, 2009; Wilcox, 2012). Increased online engagement is proposed as a remedy to issues such as low science literacy, depressed public trust in science, declining public funding of science, and poor knowledge of environmental and health issues (McClain & Neeley, 2015).

Scientists and science communicators are beginning to question the impact of online science outreach (McClain & Neeley, 2015) and both scholarly research and science communication literature indicate that online communications may suffer from a variety of issues. Practitioners often unknowingly approach online science communication with a variety of counterproductive misunderstandings and/or follow outdated models of best practices (McClain & Neeley, 2015). Engagement with online science material by social medial users is transient and brief (Thaler et al., 2012). Goals, expected outcomes, success, and evaluation are often poorly defined (Bik et al., 2015). Online science communication often occurs within ‘echo chambers’ rather than finding new audiences or audiences in the most need of science education (Williams et al., 2015). Social online capital does not necessarily translate into real-world action (Stefanone, Kwon & Lackaff, 2012; Thaler et al., 2012). Importantly, it is also unclear what science content social medial users consume, engage with, and share with others. And while it is known which aspects of general content lead to online virality and popularity (Berger & Milkman, 2012; Nelson-Field, Riebe & Newstead, 2013), what causes the same in science content is unknown.

Evidenced-based models are often lacking for online science communication, although literature quantitatively examining key questions in social media’s role in science outreach is building (Côté & Darling, 2018; Darling et al., 2013; Fauville et al., 2015; Haunschild et al., 2018; Haustein et al., 2014; McClain, 2017; Thaler et al., 2012; Thelwall et al., 2013). However, many of these studies, likely related to the accessibility of metrics and data, are focused on Twitter. This focus on Twitter is contrary to the usage and importance of other social media platforms such as Facebook.

As of November 2018, 2.23 billion people log into Facebook every month and is the world’s third most visited website (Cooper, 2018). In the U.S., 68% of the population is estimated to be on Facebook (Cooper, 2018). Daily usage rates are also exceptionally high, with 1.47 billion people (66% of Facebook users) worldwide logging in daily, which rises to 74% of U.S users (Cooper, 2018). This engagement is greater than user visitation on other social media platforms such as Instagram and Twitter (Smith, 2014). The engagement time of a user with Facebook also is traditionally longer than other social media platforms—each user spends on average 58 min a day on the site (Cooper, 2018).

The notoriety of Facebook as a source of news and information has dramatically increased since 2016, often tied to discussion of the US presidential election (Allcott & Gentzkow, 2017). Compared to other social media platforms, a higher percentage of Facebook users, 66%, also receive their news through the site (Gottfried & Shearer, 2016). This passive consumption and exposure to new topics can induce shifts in behaviors and perspectives (Messing & Westwood, 2014).

Despite the prominence of Facebook in usage and as a source of information, the platform has received less attention in science outreach than Twitter or blogging (Editorial, 2009; Bik et al., in press; Bik & Goldstein, 2013; Bonetta, 2009; Fausto et al., 2012; Fox, 2012; Goldstein, 2009; Hall, 2014; Haustein et al., 2014; Kouper, 2010; Ogden, 2013; Pinholster & Ham, 2013; Shema, Bar-Ilan & Thelwall, 2012; although see Shiffman, 2018). Indeed, few scientists believe Facebook is an effective form of online science communication (Collins, Shiffman & Rock, 2016; Fauville et al., 2015). However, recently McClain (2017) proposed that Facebook may provide an ideal platform for scientists to engage with the public because of ease of use and the more intimate and personal nature of the social networks cultivated there.

Here, I address with quantitative and predictive models a key question on the popular social media platform, Facebook. Specifically, I examine how scientific content impacts virality and engagement on a popular public Facebook page focused on marine sciences. Through an online experiment, I examine how various aspects of marine organism image content, quality, and captioning affect total likes, comments, and shares of Facebook posts.

## Methods

DSN is a popular social media and website group focused on ocean topics and science. The overall mission of DSN (http://www.deepseanews.com/about-2/) is “demystifying and humanizing science in an open conversation that instills passion, awe, and responsibility for the oceans”. DSN’s blog (http://www.deepseanews.com/) averages ∼82 K views per month and garnered ∼16 M views since December of 2006. A total of 4,134 posts have been published on the DSN blog. DSN’s Twitter account (https://twitter.com/deepseanews), in existence since 2010, currently has 17.5 K followers and nearly ∼8 K tweets. DSN’s Facebook page (https://www.facebook.com/deepseanews/), the test platform for the experiments here, was started in 2011 and currently has 17,211 followers and 17,266 likes overall (since 1/1/2017). Social medial users for the purpose of this experiment is defined as followers of DSN’s Facebook page and their Facebook friends who might also engaged with shared content. As such, the composition of the audience may bias the results, e.g., the audience is well-educated and science enthusiasts (see ‘Discussion’).

Seventy-six still images were posted to the Deep-Sea News (DSN) Facebook page between May 12, 2014 and January 12, 2016. At the close of the experiment in 2016, DSN had 14,654 followers. An average of 8 days was left between subsequent posts of images to allow for a reset period for engagement. At 24 h after posting, the total number of Facebook likes, comments, and shares were quantified. For reference, the Facebook like button allows users to show support for specific content including comments, posts, and images. The Facebook share button allows users to share the post to their own Facebook pages to share with their Facebook friends. A Facebook comment is a user remark on a post. On October 18, 2018, the current number of Facebook likes, comments, and shares were also quantified. This yielded counts at 1,000 to 1,610 days after posting. For the sake of brevity, these metrics will be referred to as current likes, comments, and shares.

Facebook posts and images were varied in the following ways:

1. *Taxonomic group featured*. Before the experiment, followers of DSN on Facebook were polled about their “favorite” taxa. This poll was simple post on DSN’s Facebook asking follower to comment about what their favorite animals or taxa were. These comments were then proofed, taxonomically standardized, and coalesced into groupings. This poll was used to limit the total number of taxonomic groups from the whole metazoan tree of life to a reduced amount appropriate for factorial analysis. That poll yielded 89 valid responses that were classified into these broad taxonomic groupings: Anthozoa (*n* = 3), Arthropoda (10), Cephalopoda (15), Cetacea (9), Echinodermata (4), Elasmobranchii (10), Gastropoda & Bivalvia (excluding Nudibranchia, 1), Medusozoa (2), Nematoda (1), Nudibranchia (6), Osteichthyes (22), Pinnipedia (1), Polychaeta (3), Porifera (1), and Testudines (1). These 15 taxonomic groups were used for the subsequent experiment.
2. *Image type*. Images were classified as either color (*n* = 26), behavioral (26), or scientific images (26). Color images were photographs of organisms in passive behavior, e.g., floating, sitting, standing. Behavioral images were denoted by the organism exhibiting an active behavior, e.g., defense, foraging, predation. Scientific images included illustrations of processes and anatomy or images of labeled specimens.
3. *Awe Factor*. Images were determined to have a high awe factor (*n* = 40, low awe factor *n* = 36) if the image quality was high, the image followed basic photographic principles; the rule of thirds, high color saturation, and/or a new/likely unknown fact relating to the organism.
4. *Caption Type*. Captions were either scientific (*n* = 33) or public (46). Science captions included heavily used jargon, scientific names, and lack of emotive text. Public captions removed jargon and included emotive text, e.g., “In our ongoing effort educate you on the parts of marine beasties. The anatomical loveliness of a coral polyp”.
5. *Caption Length.* Word count of the caption (range 5–71, mean = 27.4, median = 25.5).
6. *Date and Time of Day*. Both date and time of day were collected from each post to the Facebook page. All images were posted between 8:00 and 22:30 CST, and normally distributed between these times with a mean of 14:47 CST.

Links to all images, captions, classifications of each image based on the above criteria, and full data on likes, comments, and shares for each image post are available at https://github.com/crmcclain/facebookimageexperiment. Example images with metrics are provided in Table 1.

**Table 1:** Example images used in the study including links to the original image, link to the Facebook post, metrics for how individual images were coded, and numbers of likes, shares, and comments.

Example	**Image and post link**	**Taxa**	**Photo type**	**Caption type**	**Awe factor**	**Date**	**Caption word count**	At 24 h			Current		
								**Likes**	**Shares**	**Comments**	**Likes**	**Shares**	**Comments**
Lowest Likes	https://tinyurl.com/yd4ruyh5 https://tinyurl.com/y7ouxqcu	Pinniped	Behavioral	Public	Low	8∕3∕14	21	12	0	0	13	0	0
Second Highest Likes	https://tinyurl.com/y9pdmm6x https://tinyurl.com/ybzewpk9	Cephalopod	Color	Public	High	10∕13∕14	66	239	110	5	278	8	135
Highest Likes	https://tinyurl.com/ya4ptsna https://tinyurl.com/ydhjkcnp	Osteichthyes	Standard Scientific	Public	High	9∕18∕15	31	727	734	52	743	52	798
Examples of different factors in Echinoderms													
High Awe & Behavior	https://tinyurl.com/yamgf9z7 https://tinyurl.com/ycoog35d	Echinoderm	Behavioral	Public	High	7∕10∕15	37	49	1	0	61	0	6
High Awe & Color	https://tinyurl.com/y7g7nhtu https://tinyurl.com/y9gonbpl	Echinoderm	Color	Scientific	High	10∕16∕14	20	45	2	0	59	1	16
High Awe & Scientific	https://tinyurl.com/y98z4rjc https://tinyurl.com/y9dgp5fe	Echinoderm	Standard Scientific	Scientific	High	8∕13∕14	19	41	2	1	49	2	5
Low Awe & Color	https://tinyurl.com/y7rq99kv https://tinyurl.com/yda5df36	Echinoderm	Color	Public	Low	11∕26∕14	55	26	2	0	36	2	3
Low Awe & Scientific	https://tinyurl.com/ya7mpdum https://tinyurl.com/y8xuaglc	Echinoderm	Standard Scientific	Scientific	Low	10∕15∕14	28	26	0	2	29	2	2
Low Awe & Color	https://tinyurl.com/ya3ae2qw https://tinyurl.com/y8jrbxxx	Echinoderm	Color	Scientific	Low	8∕4∕14	17	20	0	0	21	0	2
Test post with predictive model													
	https://tinyurl.com/yaxn3zml	Cephalopod	Color	Public	High	10∕31∕18	67	231	75	13	–	–	–

All factorial combinations of categories 1–4 were calculated and post order over the experiment of these was assigned randomly. Although Facebook adjusted their newsfeed algorithm at least once during the scope of the experiment, this should not impact the results here as image post types were temporally randomly distributed. Once all factorial combinations were identified, images were searched for by taxa first on WikiMedia Commons or Flickr that met the criteria and Creative Commons license that allowed for sharing. If appropriate images were not found on either of these platforms and Google Image search for the specific taxa.

Spearman rank order correlations were calculated between Facebook likes, shares, and comments at 24 h after posting versus those for the October 8, 2018 post. Since the resulting data were counts, a Negative Binomial generalized linear model was used. Because in the case of shares and comments the data were heavily zero-inflated, a Zero-inflated Negative Binomial generalized linear model was implemented. Both of these tests were conducted in R with the *pscl* package (Jackman, 2017; Zeileis, Kleiber & Jackman, 2008). Pseudo R-squared measures were calculated for all models using the Nagelkerke method in the *rcompanion* package in R (Magiafico, 2018). Estimated marginal means (EMMs) for specified factors for the models and comparisons and contrasts among them were also calculated using the *emmeans* package (Lenth, 2018). Tukey adjustments were used for multiple comparisons.

## Results

**Figure 1:**
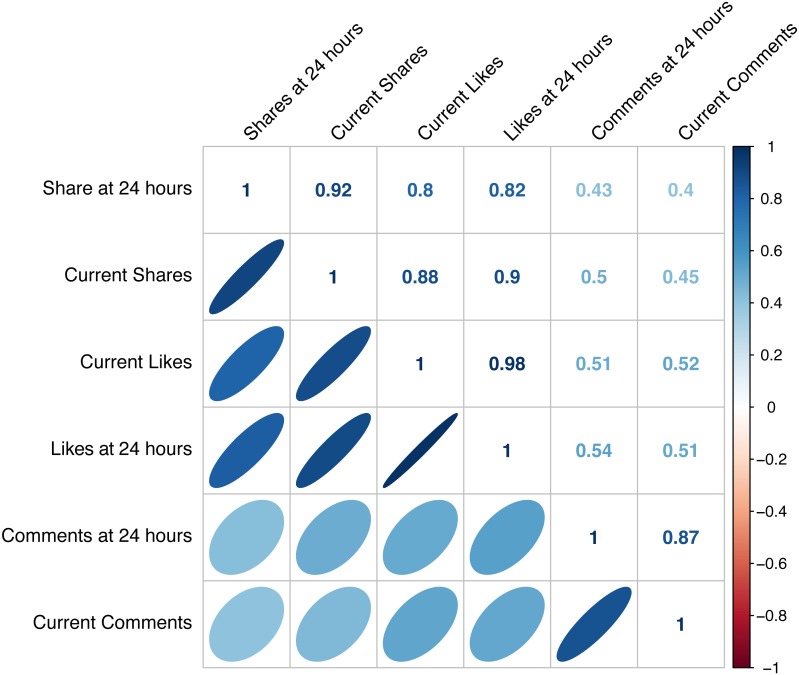
Correlation plots of likes, comments, and shares. Correlations between counts of Facebook likes, comments, and shares at 24 h after posting and at 1,000–1,610 days (current) after marine animal imagery was posted. Lower diagonal of the matrix displays correlation cloud shapes and slopes. Upper diagonal are Spearman Rank Correlation values.

Facebook likes, comments, and share counts at 24 h after posting versus counts at 1,000–1,610 days were highly correlated (Fig. 1), ranging from correlations of 0.98–0.86 (*p* ≪ 0.0001).

### Likes

**Figure 2:**
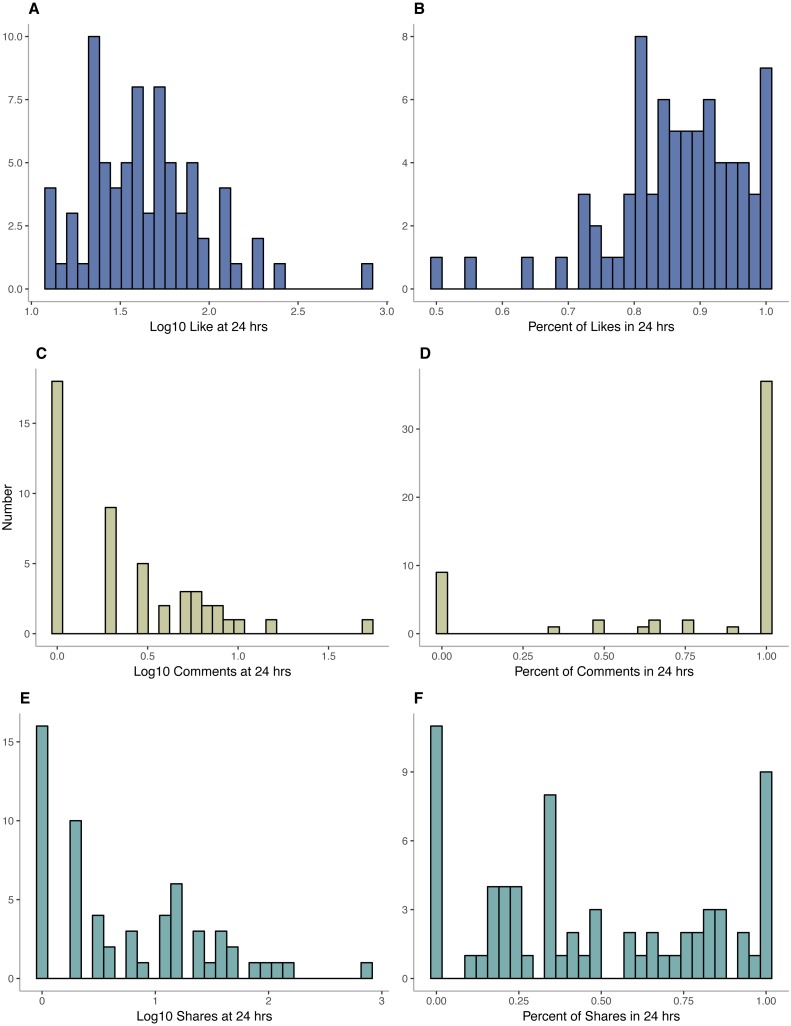
Histograms of likes, comments, and shares. Histograms of log10 likes (A), comments (C), and shares (E) at 24 h after posting of marine animal imagery. Histograms of the percentage of likes (B), comments (D), and shares (F) received in first 24 h after posting.

For likes, most activity occurred within the first 24 h after posting; 75% of image posts received 81.3% of their likes within the first day (Fig. 2). Likes within this 24-hour time span ranged from 12 to 727 (Fig. 2).

An analysis of the Facebook likes indicates the data are overdispersed with respect to a Poisson model (*p* ≪ 0.0001). A negative binomial regression was thus utilized to analyze the data. Analysis indicates that only taxa (*p* ≪ 0.0001), image type (*p* = 0.0066), and awe factor (*p* ≪ 0.0001) were significant predictors of Facebook likes on images. A full model with both significant and non-significant predictors (AIC = 725) yields a pseudo-*R*^2^ of 0.71, while the reduced model (AIC = 720) with only significant factors yielding a pseudo-*R*^2^ of 0.70.

**Figure 3:**
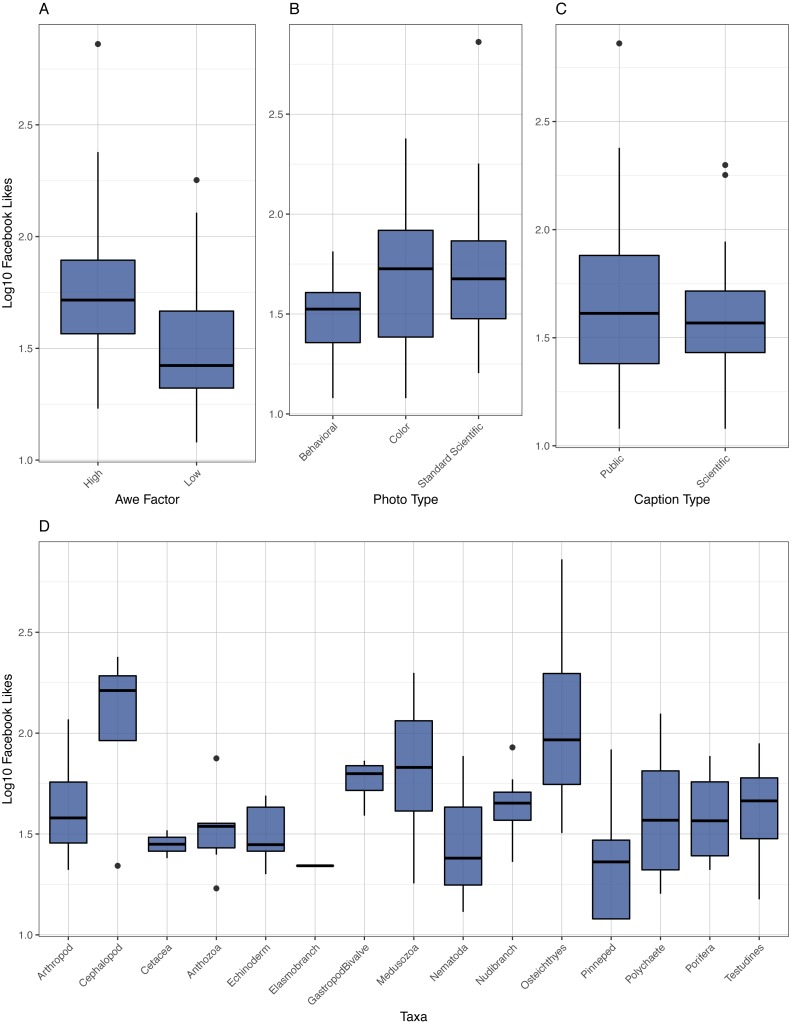
Boxplot of Likes. Boxplot of log10 Facebook likes versus awe factor, photo type, caption type, and taxa.

Images of Cephalopods and Osteichthyes generated significantly more likes that many other taxonomic groups including Echinoderms, Nematodes, Pinnipeds, and Cetaceans (*p*-value: ≫0.0001–0.0093, Fig. 3, Appendix S1). Osteichthyes also received significantly more likes than Polychaetes, Nudibranchs, Poriferans, Anthozoans, and Testudines (*p*-values: 0.0003–0.0093, Fig. 3, Appendix S1). Medusozoans also received more likes than Pinnipeds (*p* = 0.0090, Fig. 3, Appendix S1). On average, image posts of Medusozoans received 69.6, Cephalopods 115.5, and Osteichthyes 165.3 likes, whereas other taxa received between 22.3 and 56.6 likes.

Standard scientific images received significantly more likes than behavioral images (*p* = 0.0047, 55.2 versus 35.1 likes, Fig. 3, Appendix S1). Color images were not significantly different from the other two categories. Likewise, high awe images received more likes than low awe images (*p* ≫ 0.0001, 65.3 versus 33.1).

Using the predictive model for Facebook (see Appendix S1) likes and selecting for high-performing categories in taxa, awe, and photo type, an additional image was posted of a Cephalopod, in color, and with a high awe factor. The model predicted likes were 192 (range 93–290). The posted image of a bobtail squid on October 31, 2018 yielded 231 likes within 24 h after posting.

### Shares

For shares, the range spanned from 0, incorporating 21% of posts, to 734 (Fig. 2). Unlike Facebook likes, however, only 25% of posts received 80.8% of their shares within the first 24 h after posting (Fig. 2).

**Figure 4:**
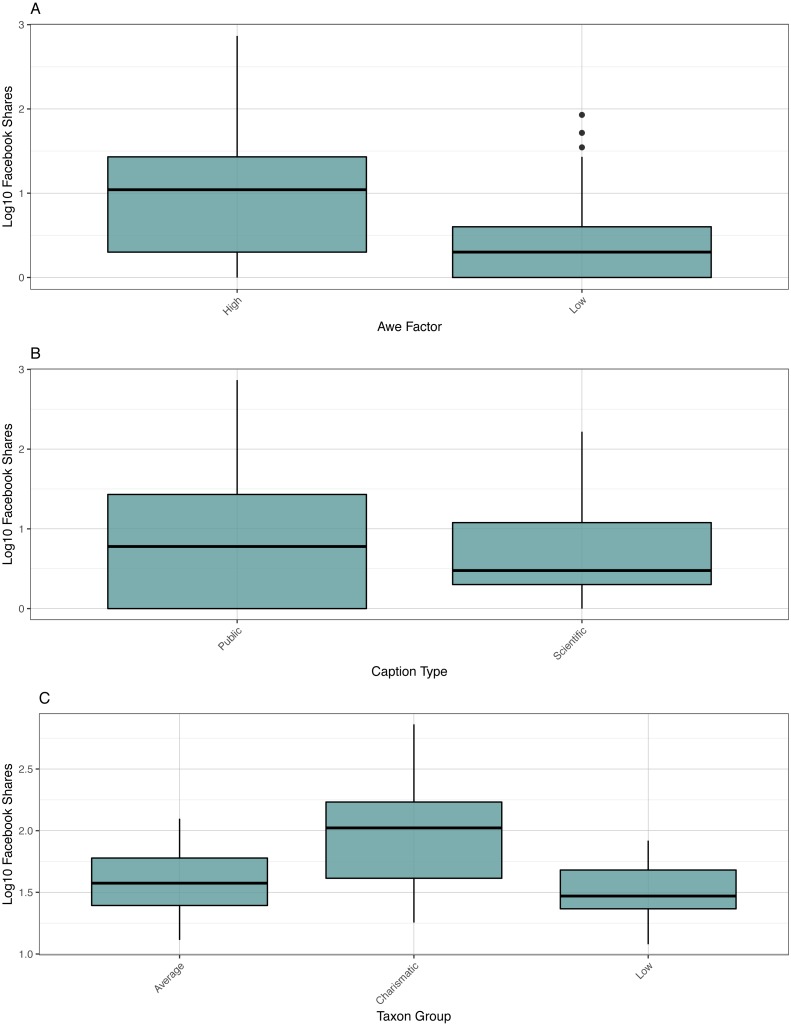
Boxplot of shares. Boxplot of log10 Facebook shares versus awe factor, caption type, and taxon group.

The data for Facebook shares on image posts was heavily zero-inflated and both Poisson (*p* = 0.0058) and negative binomial regression models (*p* ≪ 0.0001) were overdispersed. A zero-inflation negative binomial regression model was utilized to account for the distribution of the count data. Because zero-inflation models run two models on the zero and non-zero data, they are particularly sensitive to the number of states in independent variables and the number of variables. Taxa were culled into *post hoc* reduced taxonomic variable based on Facebook like performance into a low, average, and high performance category based on groups identified in pairwise comparisons. Cephalopods, Osteichthyes, and Medusozoans were placed in the high category; Gastropods/Bivalves, Elasmobranchs, Cetaceans, Echinoderms, and Pinnipeds in the low category; and the rest of the taxa placed in the average category. In addition, independent variables were not included in the share model that were nonsignificant in the Facebook like model. A Facebook share model was constructed with photo type, awe factor, caption type, and the new three-category reduced taxonomic variable. Only awe factor (*p* = 0.0013), caption type (*p* = 0.0397), and the reduced taxonomic variable (*p* ≫ 0.0001) were significant (Fig. 4, Appendix S1). This model produced a pseudo-*R*^2^ of 0.59.

Low, average, and high performance taxa received significantly different shares from each other (*p* = 0.0139-0.0423, means: 2.9, 8.2, 63.6). Although caption type was a significant factor, a Tukey post-hoc test did not yield a significant difference between shares of posts with public and scientific captions. High awe was significantly different in terms of shares from low awe images (*p* = 0.0137, 37.8 versus 12.0 average shares).

### Comments

For comments, counts ranged from 0-52, but 36.8% of posts received no comments (Fig. 2). Of the remaining posts that received comments, a majority (67.2%) received those comments in the first 24 h after posting (Fig. 2).

**Figure 5:**
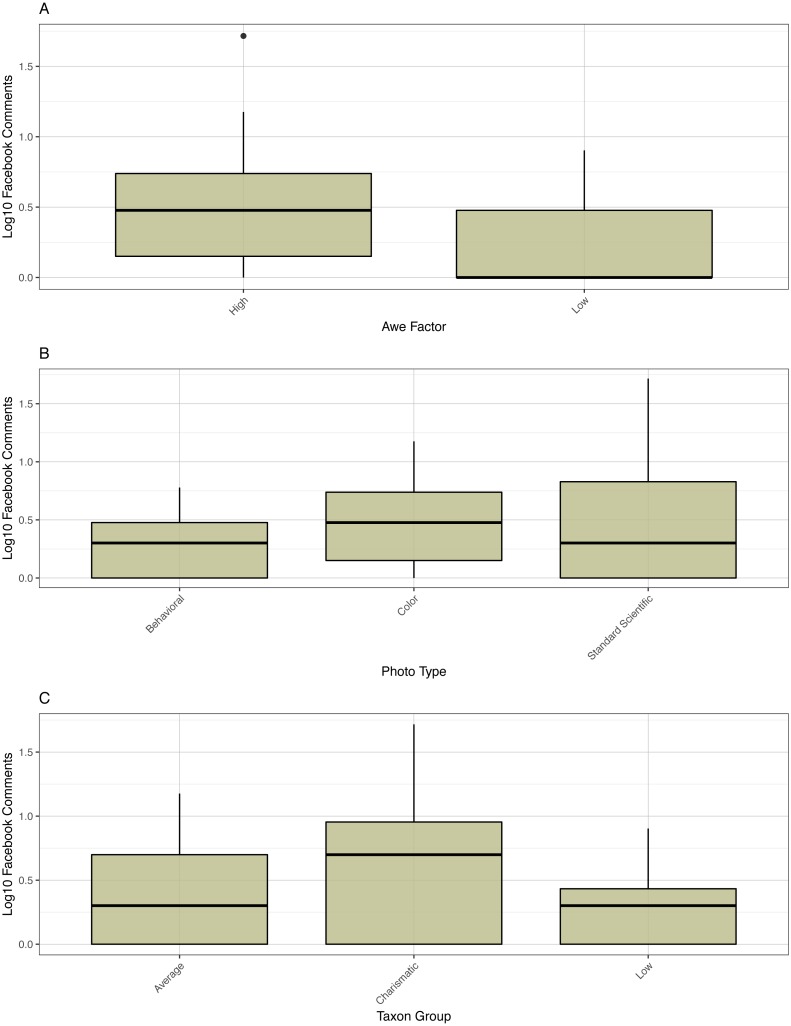
Boxplot of comments. Boxplot of log10 Facebook comments versus awe factor, photo type, and taxon group.

Facebook comment data was also heavily zero-inflated, and Poisson (*p* = 0.0053) and negative binomial regression models (*p* ≪ 0.0001) were both overdispersed. For similar reasons as the share model, the comment model was reduced to include only photo type, awe factor, caption type, and the reduced taxonomic variable, producing a pseudo-*R*^2^ of 0.32. Photo type (*p* = 0.0091), awe factor (0.0003), and the reduced taxonomic variable (0.0435) were all significant factors predicting comments but not caption type (*p* = 0.4150, Fig. 5, Appendix S1). Tukey post-hoc comparisons yielded no significant differences between different taxonomic categories or image types in terms of comments. High awe images received significantly more comments on average (3.68 versus 1.57, *p* = 0.0290).

## Discussion

### Variability in interactions

In general, the Facebook audience in the study exhibited a wide range of engagement rates with the individual posts ranging from 12 to 727 likes, 0–734 shares, and 0–52 comments. Most of this activity, especially for likes and comments, occurred within the first 24 h after posting. For all three metrics, correlations between counts at 24 h after posting versus at 1000+ days were positive, significant, and high.

### Comments

As expected, comment activity was much lower than likes and shares. In addition, many comments on the Facebook posts in this study were not commenting on the images but rather tagging another Facebook user. This pattern of liking and sharing versus commenting likely reflects the ease, a single button click, to either like or share on Facebook. The ease of superficially engaging with content has been coined “Slacktivism” and has been tied to the larger issue of “people who support a cause by performing simple measures (and) are not truly engaged or devoted to making a change” (Thaler et al., 2012). This pattern of low commenting is seen across DSN platforms; this study employs DSN’s Facebook page and the results are consistent with previous findings for the DSN blog. Previous research (Thaler et al., 2012) found that a majority of blog readers never commented (54.2%) and those that did comment left one or two comments (23.4%) over all posts they read. Surveyed readers of the blog, when asked why they did not comment, responded that they (1) did not feel qualified (28.6%), (2) had nothing to add (25.7%), or (3) did not generally comment on blogs (17.1%). Indeed, research indicates that commenting may be more heavily influenced by personality traits than social media content *per se* (Kabadayi & Price, 2014). Prior research also indicates that users’ active participation in social media, including commenting, requires practitioners to engage with users’ conversations at both the individual post and long-term levels (Camarero, Garrido & San Jose, 2018). The posts in this study were strongly visual, which may not have provided sufficient initial conversational stimulus for many users. Captions were also not specifically designed to prompt commenting, e.g., asking Facebook users a question. However, a previous study on the Facebook page of the Monterey Bay Aquarium Research Institute concluded that “an organization’s Facebook page does not seem to be a suitable space to trigger discussions” (Fauville et al., 2015).

### Shares

Share behavior was more dynamic and unpredictable than either likes or comments. The sharing of posts occurred over longer lifespans, i.e., time decay of sharing is much longer than commenting or liking. Shares were also less predictable according to the criteria measured in this study. Pseudo-*R*^2^ for the best share models was 0.32 versus 0.59 and 0.70 for comments and likes respectively. Sharing may reflect a robust social network component not captured in this study. High share counts require uptake by users with an extensive Facebook network, increasing the probability of share by their followers. The sharing network is affected by the size and structure of the Facebook user and their followers as well as positions of early sharers in the Facebook network (Weng, Menczer & Ahn, 2014). This user network effect is combined with Facebook’s content feed algorithm that weighs active interactions like commenting and sharing over passive interactions such as likes and click-throughs. Higher weighting is also assigned if users comment, share, and like the original share. To restate, shares require high activation energy but once a critical mass of shares and engagement on those shares is reached, then shared content appears more frequently in users’ feeds. This causes a positive feedback allowing other users to continue to share. Because of this activation energy required, lag times and volatility in shares may be greater. This is compounded by the fact that predicting what Facebook users share on the platform may also require knowledge of their self-interests and the perceived community incentives in their networks (Fu, Wu & Cho, 2017).

### The role of charismatic megafauna

Traditional models of “charismatic megafauna” or “flagship species” dominate conservation and science communication efforts:

The focus on species in conservation has largely centered on vertebrates especially birds and large mammals. They are visible, dominant parts of our natural environments, and, for better or worse, extract more sympathy from the public than do most plants or insects. These “flagship” or “charismatic” species draw financial support more easily than do stinging insects or obscure mussels, and by so doing serve to protect habitat and other species under the “umbrella” of their large habitat requirements (Meffe & Carroll, 1997).

In the oceans, “charismatic megafauna” generally and largely incorporates marine mammals, sharks, and to a lesser degree fish and birds (Barney, Mintzes & Yen, 2005; Gerber & Heppell, 2004; Leader-Williams & Dublin, 2000). Indeed, in a survey of what taxa were considered charismatic, sharks, dolphins, and whales were included in the top ten (Albert, Luque & Courchamp, 2018). The use of “charismatic megafauna” has been criticized, including avocation of broadening the taxa promoted in conservation and communication (Entwistle, 2000; Guerra et al., 2011). The results of this study support these criticisms by suggesting that traditional definitions of “charismatic megafauna” and what organisms the public most engage with may be incorrect. Traditional outreach knowledge suggests that “charismatic megafauna” such as pinnipeds, cetaceans, and elasmobranchs may offer increased appeal to the public. However, across likes, comments, and shares, cephalopods, osteichthyes, and medusozoans out-performed these traditionally “charismatic megafauna”. These findings suggest the concept of “charismatic megafauna” may be different from and broader than previously thought.

Further support that the concept of “charismatic megafauna” may need reexamination is that individual images of taxa often perceived as not having public appeal performed well, including images of: two gastropods and one bivalve (63, 69, 73 likes), two polychaetes (65, 125), a nematode (77), and an arthropod (117). In addition, a monthly series focused on marine gastropods that occurred in 2015 on DSN’s Facebook page on average generated 50+ likes and 20+ shares per post, often higher than the more traditionally defined “charismatic megafauna” in this study. While the ongoing gastropod series may have raised awareness of post and inflated interactions, the posts do indicate that certain methods may overcome “charismatic megafauna” bias. These results and data also suggest that while some taxa may exhibit on average lower public engagement rates online, engagement can vary considerably. In other words, key and strategic posts on “non-charismatic taxa” still have the potential for high online engagement.

### Quality and aesthetics of imagery

High-awe images consistently outperformed low-awe images by 1.6–3.2 times in likes, comments, and shares on Facebook. This suggests careful selection and curation of imagery is vital for online science engagement. Indeed, aesthetics contribute significantly to online engagement with image-based posts, including aspects of color, spatial arrangement, and complexity (Schifanella, Redi & Aiello, 2015). These results are also consistent with other research demonstrating that *New York Times* articles that connect emotionally with readers are more likely to be engaged with through comments, shares, and likes (Berger & Milkman, 2012). Specifically, content that evokes high physiological arousal, i.e., awe; a positive rather than a negative reaction; or an emotive reaction generates more engagement (Berger & Milkman, 2012; Kim, 2015; Nelson-Field, Riebe & Newstead, 2013). Awe while encompassing aspects of emotion may also incorporate novelty, and the combination of these are known to bolster content virality on Twitter (Vosoughi, Roy & Aral, 2018). For example, standard scientific images, with increased likes, often exhibited unknown animal features to Facebook users, i.e., novelty through informational discovery. The better performance of high-awe images in this study may benefit from both increased positive and emotive reaction as well as novelty. An image post of *Mola mola*, or sunfish, internal anatomy detailing the unique musculature and skeleton of the animal received the most number of likes and shares of all images deployed in the experiment: 727 and 734, respectively, in the first 24 h after posting.

### Audience biases

The established audience of the DSN’s Facebook page, where this experiment occurred, could have impacted the generality of the results. Gender and age group are known to affect online engagement with specific content (Han et al., 2018). Privacy standards of Facebook prevent knowing the demographic composition of DSN’s Facebook followers. Although the overlap and similarities between the audience of DSN’s blog and Facebook page are unclear, biases in the demographics of the blog readership may also exist for the Facebook page. A prior survey of the DSN blog may provide some insights (Thaler et al., 2012). The typical DSN blog reader is a American young adult (25–34 years old; 41.5%) or middle-aged individual (35–54 years old; 34.5%). Readership was slightly more male (51.5%) and largely from the United States and Canada, followed by the United Kingdom (70.3%, 9.0%, and 4.0% respectively). The DSN blogs has a well-educated readership with a high percentage of readers having or pursuing a graduate or professional degree (17%). If these statistics hold for DSN’s Facebook audience as well then certain biases may affect the results. For example, the overall education of the audience and insular effect of attracting an audience with interests in the oceans may result in increased interest in a variety of taxa, ameliorating the “charismatic megafauna” effect.

## Conclusions

Imagery is an essential component of the social media landscape. The inclusion of imagery is important for audience engagement with posts on Facebook (Su, Reynolds & Sun, 2015). However, little is known about how online audiences engage with image content and share it with their networks (Bakhshi, Shamma & Gilbert, 2014). This lack of knowledge applies equally to scientific imagery, a vital component of conveying information both in scientific inreach and outreach. Here, I show that a basic quantitative model can be useful in predicting response to marine organism imagery on Facebook. While the “charismatic megafauna” effect exists, i.e., audiences engage with imagery of some taxa more than others, this effect is counter to traditional views of what can be classed as “charismatic megafauna” and suggests a high degree of variability in what taxa the public connect with. Thus, science communicators should be more cautious in the choice and exclusion of organisms to include in outreach efforts. The results also demonstrate that imagery that presented novel information and was aesthetically pleasing received on average 2× more likes and shares than other posts. The findings here indicate that investment by science communicators in high-quality imagery and professional photographers and artists may greatly improve outreach efforts. Aspects of text including the length of the caption and amount of jargon did not significantly impact either likes, shares, or comments a post received and the virality of scientific images may not be related to textual explanation. Furthermore, audiences may not be engaging with captions and information that is intended to be conveyed to the user should be contained in the image itself. This study provides one of the first quantitative analysis of virility of scientific images via social media and challenges previously held conceptions of best practices of social media scientific outreach.

##  Supplemental Information

10.7717/peerj.6795/supp-1 Appendix S1 Appendix R Package Code and Results from analyses.

## Additional Information and Declarations

### Data Availability

The following information was supplied regarding data availability: Data is available at: https://github.com/crmcclain/facebookimageexperiment.
