# Supplementary material for: Likes, comments, and shares of marine organism imagery on Facebook
Source: PeerJ. 2019 Apr 24;7:e6795. doi: 10.7717/peerj.6795 (PMC6486811; doi:10.7717/peerj.6795)
Supplement: Appendix S1 — R Package Code and Results from analyses. [file peerj-07-6795-s001.pdf]

Facebook Photo Analysis

Craig R. McClain

12/12/18

Packages Needed

```
require(ggplot2)
require(dplyr)
library(chron)
library(gridExtra)
require(DescTools)
library(car)
library(scales)
require(corrplot)
library(Hmisc)
library(plyr)
library(AER)
library(MASS)
library(rcompanion)
library(multcompView)
library(emmeans)
library(pscl)
library(knitr)
library(kableExtra)
```

Load and Process Data

```
setwd("~/Desktop/Facebook Paper/Facebook Experiment")
fb_photo<-read.csv("taxa_experiment.csv", header=TRUE)
glimpse(fb_photo)
```

```
## Observations: 76
## Variables: 13
## $ Taxa            <fct> Gastropod  Non nudibranch  & Bivalve, Jellyfis...
## $ Photo.Type      <fct> Standard Scientific, Standard Scientific, Colo...
## $ Caption.Type    <fct> Public, Scientific, Public, Scientific, Public...
## $ Awe.Factor      <fct> Low, Low, High, High, High, High, Low, Low, Lo...
## $ Date            <fct> 6/17/15, 5/13/15, 7/3/14, 12/9/14, 6/12/15, 7/...
## $ Time            <fct> 12:39:00, 21:40:00, 8:04:00, 15:58:00, 12:11:0...
## $ Like.24         <int> 69, 88, 126, 77, 148, 59, 12, 18, 15, 85, 12, ...
## $ Share.24        <int> 27, 17, 42, 13, 45, 17, 0, 1, 1, 15, 0, 0, 0, ...
## $ Comment.24      <int> 8, 0, 0, 0, 9, 3, 0, 1, 0, 2, 1, 0, 0, 1, 0, 0...
## $ Word.Count      <int> 26, 5, 25, 13, 30, 11, 21, 18, 29, 31, 12, 17,...
## $ Like.Current    <int> 78, 91, 137, 91, 150, 60, 13, 22, 20, 91, 19, ...
## $ Comment.Current <int> 9, 0, 0, 0, 9, 3, 0, 1, 0, 2, 1, 1, 3, 1, 1, 2...
## $ Share.Current   <int> 27, 17, 42, 13, 45, 17, 0, 1, 1, 15, 0, 0, 0, ...
```

```
fb_data <- fb_photo %>%
  mutate(Date = as.Date(Date , "%m/%d/%y"),
         Time = chron(times = Time),
         Days = as.numeric(Date-min(Date)),
         LogLikes = log10(Like.24),
         LogShares = log10(Share.24),
         LogComments = log10(Comment.24),
         LogCapCount = log10(Word.Count),
         Percent.Likes = Like.24/Like.Current,
         Percent.Comment = Comment.24/Comment.Current,
         Percent.Share = Share.24/Share.Current
  )

fb_data$Taxa <- revalue(fb_data$Taxa, c("Jellyfish"="Medusozoa", "Coral" = "Anthozoa","G
astropod  Non nudibranch  & Bivalve"="GastropodBivalve"))

glimpse(fb_data)
```

```
## Observations: 76
## Variables: 21
## $ Taxa            <fct> GastropodBivalve, Medusozoa, Medusozoa, Porife...
## $ Photo.Type      <fct> Standard Scientific, Standard Scientific, Colo...
## $ Caption.Type    <fct> Public, Scientific, Public, Scientific, Public...
## $ Awe.Factor      <fct> Low, Low, High, High, High, High, Low, Low, Lo...
## $ Date            <date> 2015-06-17, 2015-05-13, 2014-07-03, 2014-12-0...
## $ Time            <S3: times> 12:39:00, 21:40:00, 08:04:00, 15:58:00, ...
## $ Like.24         <int> 69, 88, 126, 77, 148, 59, 12, 18, 15, 85, 12, ...
## $ Share.24        <int> 27, 17, 42, 13, 45, 17, 0, 1, 1, 15, 0, 0, 0, ...
## $ Comment.24      <int> 8, 0, 0, 0, 9, 3, 0, 1, 0, 2, 1, 0, 0, 1, 0, 0...
## $ Word.Count      <int> 26, 5, 25, 13, 30, 11, 21, 18, 29, 31, 12, 17,...
## $ Like.Current    <int> 78, 91, 137, 91, 150, 60, 13, 22, 20, 91, 19, ...
## $ Comment.Current <int> 9, 0, 0, 0, 9, 3, 0, 1, 0, 2, 1, 1, 3, 1, 1, 2...
## $ Share.Current   <int> 27, 17, 42, 13, 45, 17, 0, 1, 1, 15, 0, 0, 0, ...
## $ Days            <dbl> 401, 366, 52, 211, 396, 58, 83, 437, 101, 155,...
## $ LogLikes        <dbl> 1.838849, 1.944483, 2.100371, 1.886491, 2.1702...
## $ LogShares       <dbl> 1.4313638, 1.2304489, 1.6232493, 1.1139434, 1....
## $ LogComments     <dbl> 0.9030900, -Inf, -Inf, -Inf, 0.9542425, 0.4771...
## $ LogCapCount     <dbl> 1.4149733, 0.6989700, 1.3979400, 1.1139434, 1....
## $ Percent.Likes   <dbl> 0.8846154, 0.9670330, 0.9197080, 0.8461538, 0....
## $ Percent.Comment <dbl> 0.8888889, NaN, NaN, NaN, 1.0000000, 1.0000000...
## $ Percent.Share   <dbl> 1.0000000, 1.0000000, 1.0000000, 1.0000000, 1....
```

Color Pallettes

Likes #3b5998 Shares #5B9A9B Comments #BDBC89

Correlations Between Old and New Data

```
l1<- ggplot(fb_data, aes(x=log10(Like.24), y=log10(Like.Current)))+
  geom_point(pch=21, size=3, fill="#3b5998", color="black", alpha=.8)+
  geom_abline(intercept=0, slope=1)+
  geom_abline(intercept=.1, slope=1, linetype=2)+
  xlab("Log10 Likes at 24 hrs")+
  ylab("Log10 Likes Current")+
  ggtitle("A")+
  theme_bw(base_size=10)+
  theme(axis.line = element_line(colour = "darkgrey"),
        panel.grid.major = element_blank(),
        panel.grid.minor = element_blank(),
        panel.border = element_blank(),
        legend.position="none",
        plot.title = element_text(lineheight=.8, face="bold", hjust = 0))



c1 <- ggplot(fb_data, aes(x=log10(Comment.24), y=log10(Comment.Current)))+
  geom_jitter(pch=21, size=3, fill="#BDBC89", color="black",
              alpha=.8, width = 0.02)+
  geom_abline(intercept=0, slope=1)+
  geom_abline(intercept=.1, slope=1, linetype=2)+
  xlab("Log10 Comments at 24 hrs")+
  ylab("Log10 Comments Current")+
  ggtitle("B")+
  theme_bw(base_size=10)+
  theme(axis.line = element_line(colour = "darkgrey"),
        panel.grid.major = element_blank(),
        panel.grid.minor = element_blank(),
        panel.border = element_blank(),
        legend.position="none",
        plot.title = element_text(lineheight=.8, face="bold", hjust = 0))

s1 <- ggplot(fb_data, aes(x=log10(Share.24), y=log10(Share.Current)))+
  geom_jitter(pch=21, size=3, fill="#5B9A9B", color="black",
              alpha=.8, width = 0.02)+
  geom_abline(intercept=0, slope=1)+
  geom_abline(intercept=.1, slope=1, linetype=2)+
  xlab("Log10 Shares at 24 hrs")+
  ylab("Log10 Shares Current")+
  ggtitle("C")+
  theme_bw(base_size=10)+
  theme(axis.line = element_line(colour = "darkgrey"),
        panel.grid.major = element_blank(),
        panel.grid.minor = element_blank(),
        panel.border = element_blank(),
        legend.position="none",
        plot.title = element_text(lineheight=.8, face="bold", hjust = 0))

grid.arrange(l1,c1,s1, ncol=1)
```

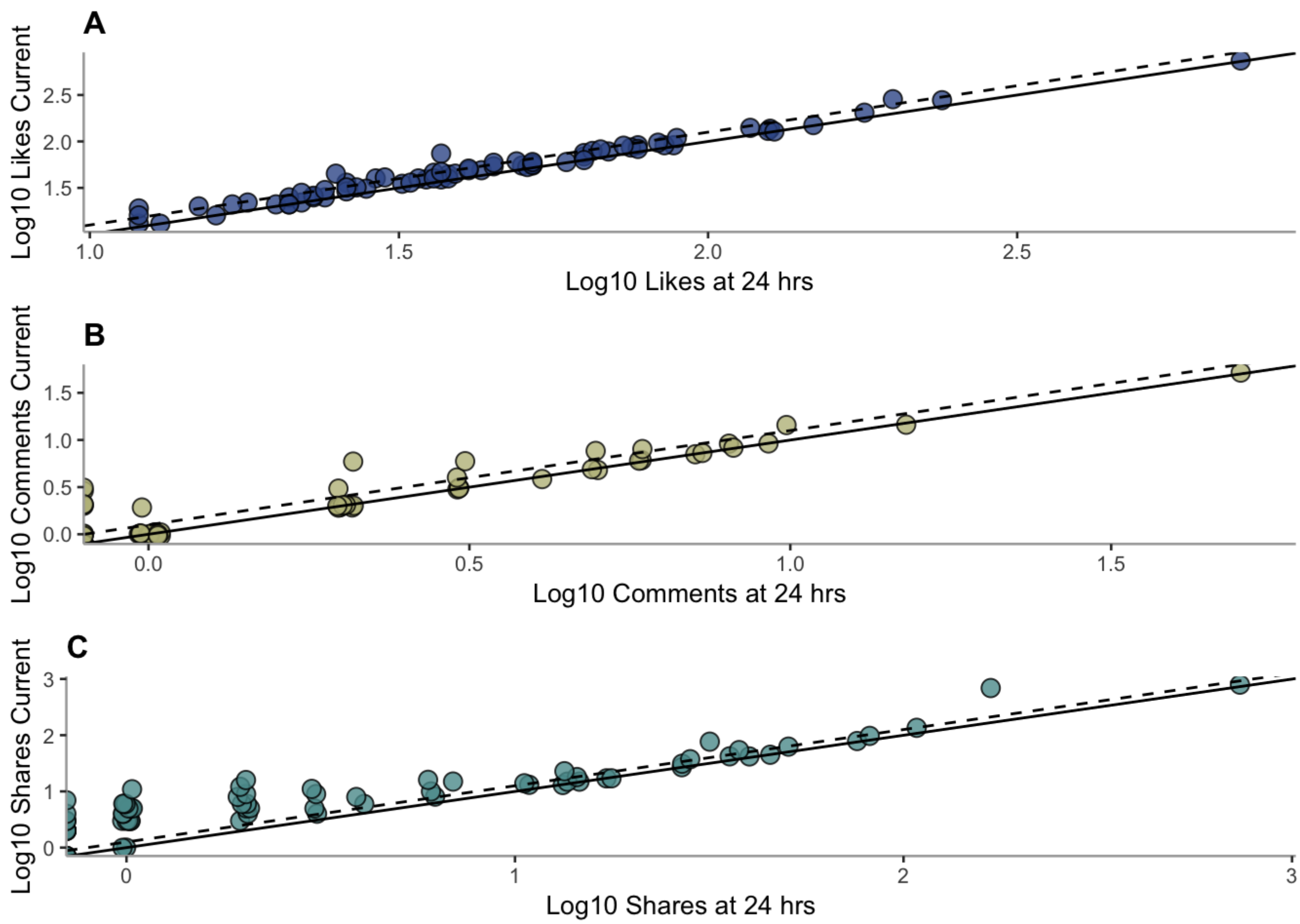

Correlation Analyses

```
fb_corr <- fb_data %>%
  dplyr::select(Like.24, Share.24, Comment.24, Like.Current, Comment.Current,
         Share.Current)

M <- cor(fb_corr,use="complete.obs", method="spearman")
kable(M) %>%
  kable_styling(bootstrap_options = c("striped", "hover", "condensed"))
```

	Like.24	Share.24	Comment.24	Like.Current	Comment.Current	Share.Current
Like.24	1.0000000	0.8223036	0.5425386	0.9823670	0.5141095	0.8967857
Share.24	0.8223036	1.0000000	0.4286272	0.8029587	0.4034404	0.9156881
Comment.24	0.5425386	0.4286272	1.0000000	0.5071848	0.8656934	0.4975335
Like.Current	0.9823670	0.8029587	0.5071848	1.0000000	0.5213614	0.8811724
Comment.Current	0.5141095	0.4034404	0.8656934	0.5213614	1.0000000	0.4482398
Share.Current	0.8967857	0.9156881	0.4975335	0.8811724	0.4482398	1.0000000

```
rcorr(as.matrix(fb_corr), type="spearman")
```

```
##                 Like.24 Share.24 Comment.24 Like.Current Comment.Current
## Like.24            1.00     0.83       0.54         0.98            0.51
## Share.24           0.83     1.00       0.43         0.80            0.40
## Comment.24         0.54     0.43       1.00         0.51            0.87
## Like.Current       0.98     0.80       0.51         1.00            0.52
## Comment.Current    0.51     0.40       0.87         0.52            1.00
## Share.Current      0.90     0.92       0.50         0.88            0.45
##                 Share.Current
## Like.24                  0.90
## Share.24                 0.92
## Comment.24               0.50
## Like.Current             0.88
## Comment.Current          0.45
## Share.Current            1.00
##
## n
##                 Like.24 Share.24 Comment.24 Like.Current Comment.Current
## Like.24              76       76         76           74              74
## Share.24             76       76         76           74              74
## Comment.24           76       76         76           74              74
## Like.Current         74       74         74           74              74
## Comment.Current      74       74         74           74              74
## Share.Current        74       74         74           74              74
##                 Share.Current
## Like.24                    74
## Share.24                   74
## Comment.24                 74
## Like.Current               74
## Comment.Current            74
## Share.Current              74
##
## P
##                 Like.24 Share.24 Comment.24 Like.Current Comment.Current
## Like.24                 0e+00    0e+00      0e+00        0e+00
## Share.24        0e+00            1e-04      0e+00        4e-04
## Comment.24      0e+00   1e-04               0e+00        0e+00
## Like.Current    0e+00   0e+00    0e+00                   0e+00
## Comment.Current 0e+00   4e-04    0e+00      0e+00
## Share.Current   0e+00   0e+00    0e+00      0e+00        0e+00
##                 Share.Current
## Like.24         0e+00
## Share.24        0e+00
## Comment.24      0e+00
## Like.Current    0e+00
## Comment.Current 0e+00
## Share.Current
```

```
quantile(fb_data$Percent.Likes, na.rm=TRUE)
```

```
##        0%       25%       50%       75%      100%
## 0.5000000 0.8135034 0.8747720 0.9326923 1.0000000
```

```r
quantile(fb_data$Like.24, na.rm=TRUE)
```

```
##     0%    25%    50%    75%   100% 
##  12.00  24.75  38.50  65.50 727.00
```

```r
summary(fb_data$Percent.Comment)
```

```
##    Min. 1st Qu.  Median    Mean 3rd Qu.    Max.    NA's 
##  0.0000  0.6667  1.0000  0.7760  1.0000  1.0000      21
```

```r
summary(fb_data$Comment.24)
```

```
##    Min. 1st Qu.  Median    Mean 3rd Qu.    Max. 
##   0.000   0.000   1.000   2.737   3.000  52.000
```

```r
sum(fb_data$Comment.24 == 0)/76
```

```
## [1] 0.3684211
```

```r
sum(fb_data$Percent.Comment== 1, na.rm=TRUE)/(76-21)
```

```
## [1] 0.6727273
```

```r
quantile(fb_data$Percent.Comment, na.rm=TRUE)
```

```
##        0%       25%       50%       75%      100% 
## 0.0000000 0.6666667 1.0000000 1.0000000 1.0000000
```

```r
summary(fb_data$Share.24)
```

```
##    Min. 1st Qu.  Median    Mean 3rd Qu.    Max. 
##    0.00    1.00    2.00   22.55   14.00  734.00
```

```r
sum(fb_data$Share.24 == 0)/76
```

```
## [1] 0.2105263
```

```r
summary(fb_data$Percent.Share)
```

```
##    Min. 1st Qu.  Median    Mean 3rd Qu.    Max.    NA's 
##  0.0000  0.2000  0.3875  0.4748  0.8075  1.0000       6
```

```
l2 <- ggplot(fb_data, aes(log10(Like.24)))+
  geom_histogram(fill="#3b5998", color="black", alpha=.8)+
  theme_bw(base_size=10)+
  ylab(NULL)+
  xlab("Log10 Like at 24 hrs")+
  theme(axis.line = element_line(colour = "darkgrey"),
        panel.grid.major = element_blank(),
        panel.grid.minor = element_blank(),
        panel.border = element_blank(),
        legend.position="none",
        plot.title = element_text(lineheight=.8, face="bold", hjust = 0))


c2 <- ggplot(fb_data, aes(log10(Comment.24)))+
  geom_histogram(fill="#BDBC89", color="black", alpha=.8)+
  theme_bw(base_size=10)+
  ylab("Number")+
  xlab("Log10 Comments at 24 hrs")+
  theme(axis.line = element_line(colour = "darkgrey"),
        panel.grid.major = element_blank(),
        panel.grid.minor = element_blank(),
        panel.border = element_blank(),
        legend.position="none",
        plot.title = element_text(lineheight=.8, face="bold", hjust = 0))

s2 <- ggplot(fb_data, aes(log10(Share.24)))+
  geom_histogram(fill="#5B9A9B", color="black", alpha=.8)+
  theme_bw(base_size=10)+
  ylab(NULL)+
  xlab("Log10 Shares at 24 hrs")+
  theme(axis.line = element_line(colour = "darkgrey"),
        panel.grid.major = element_blank(),
        panel.grid.minor = element_blank(),
        panel.border = element_blank(),
        legend.position="none",
        plot.title = element_text(lineheight=.8, face="bold", hjust = 0))

l2b <- ggplot(fb_data, aes(x=Percent.Likes))+
  geom_histogram(fill="#3b5998", color="black", alpha=.8)+
  theme_bw(base_size=10)+
  ylab(NULL)+
  xlab("Percent of Likes in 24 hrs")+
  theme(axis.line = element_line(colour = "darkgrey"),
        panel.grid.major = element_blank(),
        panel.grid.minor = element_blank(),
        panel.border = element_blank(),
        legend.position="none",
        plot.title = element_text(lineheight=.8, face="bold", hjust = 0))


c2b <- ggplot(fb_data, aes(x=Percent.Comment))+
  geom_histogram(fill="#BDBC89", color="black", alpha=.8)+
  theme_bw(base_size=10)+
```

```
  ylab(NULL)+
  xlab("Percent of Comments in 24 hrs")+
  theme(axis.line = element_line(colour = "darkgrey"),
        panel.grid.major = element_blank(),
        panel.grid.minor = element_blank(),
        panel.border = element_blank(),
        legend.position="none",
        plot.title = element_text(lineheight=.8, face="bold", hjust = 0))

s2b <- ggplot(fb_data, aes(Percent.Share))+
  geom_histogram(fill="#5B9A9B", color="black", alpha=.8)+
  theme_bw(base_size=10)+
  ylab(NULL)+
  xlab("Percent of Shares in 24 hrs")+
  theme(axis.line = element_line(colour = "darkgrey"),
        panel.grid.major = element_blank(),
        panel.grid.minor = element_blank(),
        panel.border = element_blank(),
        legend.position="none",
        plot.title = element_text(lineheight=.8, face="bold", hjust = 0))

grid.arrange(l2, l2b, c2, c2b, s2, s2b, ncol=2)
```

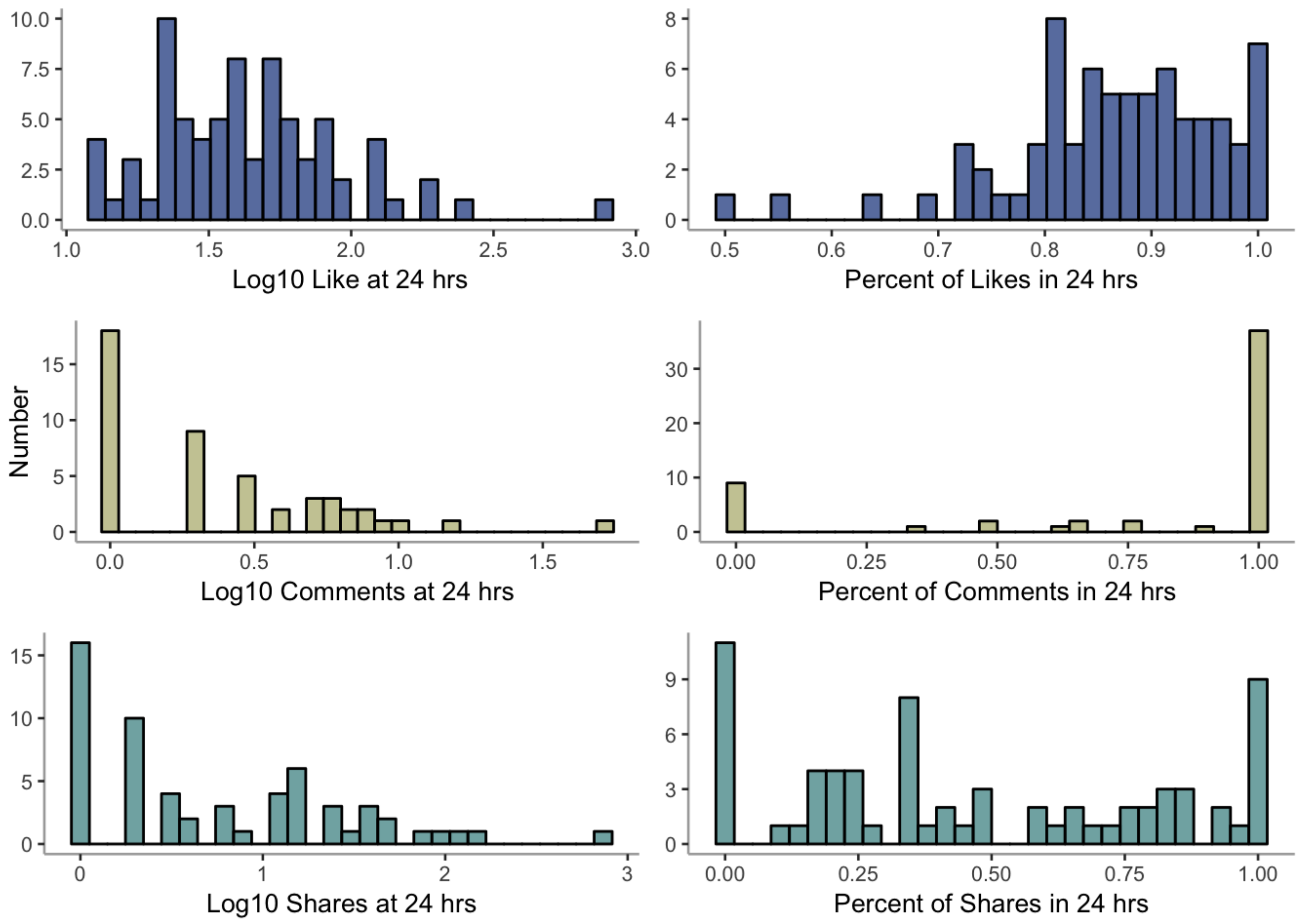

Predicting Likes Analyses

```
p1 <- ggplot(fb_data, aes(Awe.Factor,LogLikes))+
        geom_boxplot(fill="#3b5998", color="black", alpha=.8, notch=FALSE)+
        theme_bw(base_size=10)+
        theme(panel.grid.major = element_line(size = .2, color = "grey"))+
        xlab("Awe Factor")+
        ylab("Log10 Facebook Likes")+
        theme(axis.text.x = element_text(angle = 45, hjust = 1))

p2 <- ggplot(fb_data, aes(Photo.Type, LogLikes))+
        geom_boxplot(fill="#3b5998", color="black", alpha=.8, notch=FALSE)+
        theme_bw(base_size=10)+
        theme(panel.grid.major = element_line(size = .2, color = "grey"))+
        xlab("Photo Type")+
        ylab(NULL) +
        theme(axis.text.x = element_text(angle = 45, hjust = 1))

p3 <- ggplot(fb_data, aes(Caption.Type, LogLikes))+
        geom_boxplot(fill="#3b5998", color="black", alpha=.8, notch=FALSE)+
        theme_bw(base_size=10)+
        theme(panel.grid.major = element_line(size = .2, color = "grey"))+
        xlab("Caption Type")+
        ylab(NULL)+
        theme(axis.text.x = element_text(angle = 45, hjust = 1))


p4 <- ggplot(fb_data, aes(Taxa, LogLikes))+
        geom_boxplot(fill="#3b5998", color="black", alpha=.8, notch=FALSE)+
        theme_bw(base_size=10)+
        theme(panel.grid.major = element_line(size = .2, color = "grey"))+
        xlab("Taxa")+
        ylab("Log10 Facebook Likes")+
        theme(axis.text.x = element_text(angle = 45, hjust = 1))+
        scale_x_discrete(labels = wrap_format(15))

grid.arrange(p1,p2,p3,p4, layout_matrix = rbind(c(1, 2, 3),
                                                c(4, 4, 4)))
```

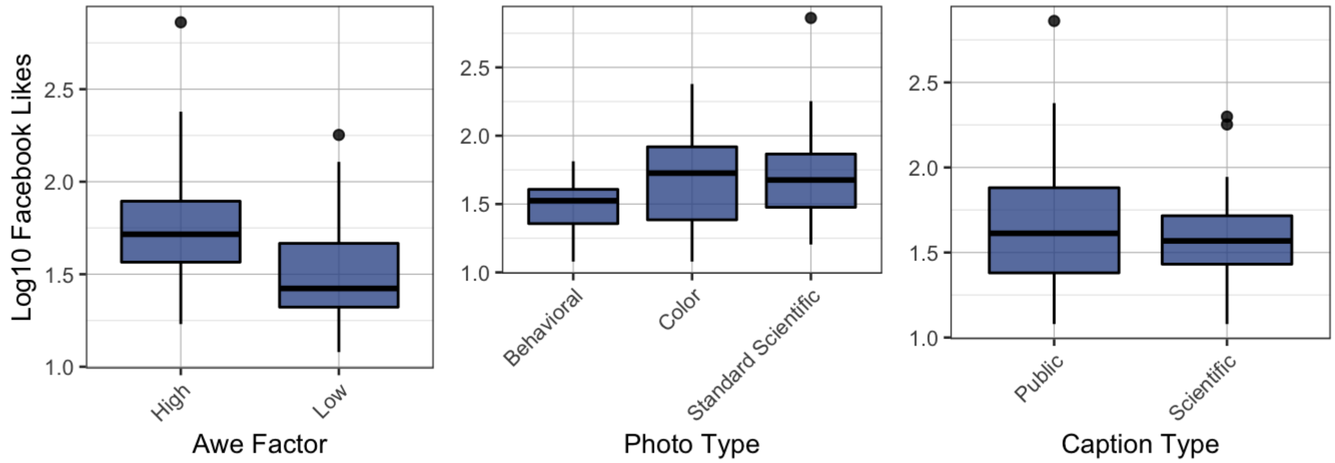

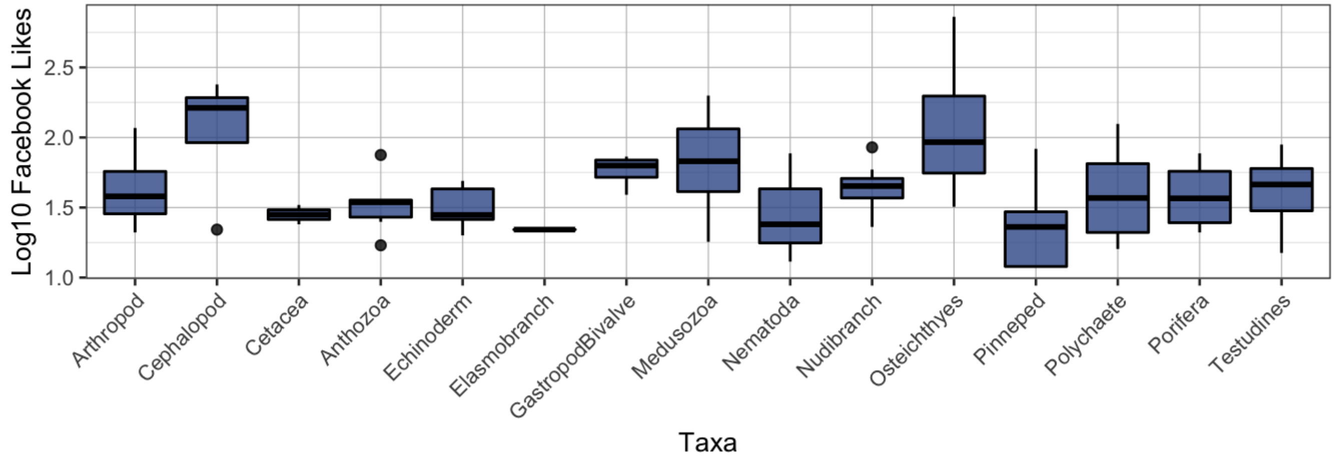

Predicting Likes Analyses

```
hist(fb_data$Like.24,breaks=50)
```

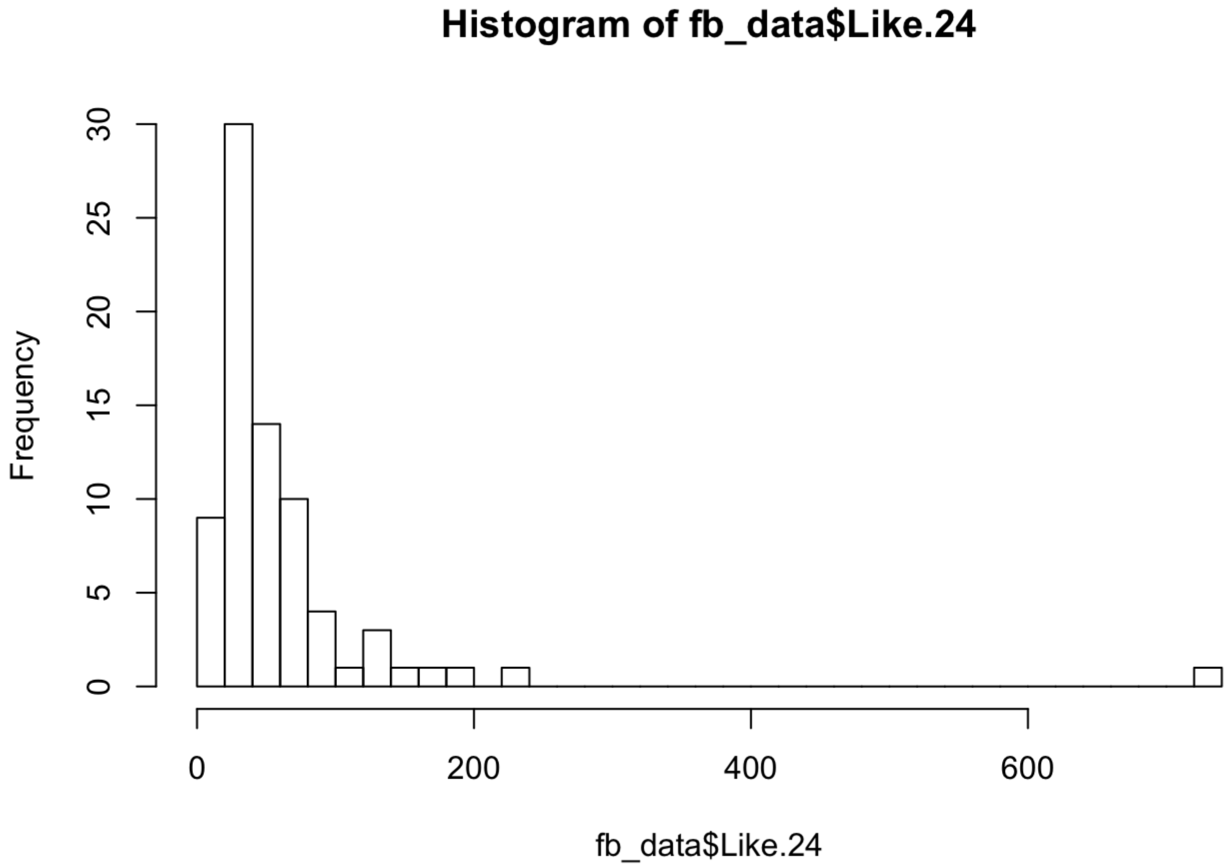

Count data so will need to use Poisson model and test for overdispersion

```
like_model_poisson<-glm(data=fb_data, Like.24~
                     Caption.Type+
                     Taxa+
                     Photo.Type+
                     Awe.Factor+
                     Days+
                     Time+
                     LogCapCount, family=poisson)
dispersiontest(like_model_poisson,trafo=1)
```

```
##
##  Overdispersion test
##
## data:  like_model_poisson
## z = 5.4013, p-value = 3.308e-08
## alternative hypothesis: true alpha is greater than 0
## sample estimates:
##   alpha
## 13.4819
```

Model is over diserpsed with p-value = 3.308e-08. Proceeding with Negative binomial regression

```r
like_model_negbin = glm.nb(data=fb_data, Like.24~
                              Caption.Type+
                              Taxa+
                              Photo.Type+
                              Awe.Factor+
                              Days+
                              Time+
                              LogCapCount,
                    control = glm.control(maxit=10000))
  #anova
  Anova(like_model_negbin,
        type="II",
        test="LR")
```

```
## Analysis of Deviance Table (Type II tests)
##
## Response: Like.24
##              LR Chisq Df Pr(>Chisq)
## Caption.Type    0.891  1   0.345106
## Taxa           82.921 14  8.099e-12 ***
## Photo.Type     10.037  2   0.006614 **
## Awe.Factor     31.686  1  1.813e-08 ***
## Days            1.122  1   0.289535
## Time            0.004  1   0.949996
## LogCapCount     0.064  1   0.799995
## ---
## Signif. codes:  0 '***' 0.001 '**' 0.01 '*' 0.05 '.' 0.1 ' ' 1
```

```r
  #pseudo r squared
  nagelkerke(like_model_negbin)
```

```
## $Models
##

## Model: "glm.nb, Like.24 ~ Caption.Type + Taxa + Photo.Type + Awe.Factor + Days + Time
+ LogCapCount, fb_data, glm.control(maxit = 10000), 4.936371911, log"
## Null:  "glm.nb, Like.24 ~ 1, fb_data, glm.control(maxit = 10000), 1.535934784, log"

##
## $Pseudo.R.squared.for.model.vs.null
##                              Pseudo.R.squared
## McFadden                             0.121142
## Cox and Snell (ML)                   0.708538
## Nagelkerke (Cragg and Uhler)         0.708565
##
## $Likelihood.ratio.test
##  Df.diff LogLik.diff  Chisq    p.value
##      -21     -46.848 93.696 3.6897e-11
##
## $Number.of.observations
##
## Model: 76
## Null:  76
##
## $Messages
## [1] "Note: For models fit with REML, these statistics are based on refitting with ML"
##
## $Warnings
## [1] "None"
```

```
 #comparisons and contrasts
  marginal_taxa = emmeans(like_model_negbin, ~ Taxa )

  kable(pairs(marginal_taxa, adjust="tukey"))  %>%
  kable_styling(bootstrap_options = c("striped", "hover", "condensed"))
```

contrast	estimate	SE	df	z.ratio	p.value
Arthropod - Cephalopod	-0.7136805	0.3018141	Inf	-2.3646362	0.5414825
Arthropod - Cetacea	0.9322698	0.4110606	Inf	2.2679622	0.6146610
Arthropod - Anthozoa	0.3665847	0.2726988	Inf	1.3442844	0.9915361
Arthropod - Echinoderm	0.5653600	0.2637377	Inf	2.1436447	0.7052877
Arthropod - Elasmobranch	0.2057118	0.5425673	Inf	0.3791453	1.0000000
Arthropod - GastropodBivalve	0.0479444	0.2859670	Inf	0.1676570	1.0000000
Arthropod - Medusozoa	-0.2073153	0.2745779	Inf	-0.7550329	0.9999880
Arthropod - Nematoda	0.8772193	0.3445237	Inf	2.5461797	0.4070057
Arthropod - Nudibranch	0.2573824	0.2552839	Inf	1.0082202	0.9996085

contrast	estimate	SE	df	z.ratio	p.value
Arthropod - Osteichthyes	-1.0724220	0.3026880	Inf	-3.5429953	0.0304265
Arthropod - Pinneped	0.8333020	0.2751042	Inf	3.0290417	0.1421629
Arthropod - Polychaete	0.3712654	0.2878591	Inf	1.2897470	0.9943841
Arthropod - Porifera	0.3481488	0.3180141	Inf	1.0947590	0.9990089
Arthropod - Testudines	0.1306322	0.2788734	Inf	0.4684284	1.0000000
Cephalopod - Cetacea	1.6459503	0.4251116	Inf	3.8718072	0.0092959
Cephalopod - Anthozoa	1.0802652	0.3052647	Inf	3.5387820	0.0308628
Cephalopod - Echinoderm	1.2790405	0.3068695	Inf	4.1680274	0.0028335
Cephalopod - Elasmobranch	0.9193923	0.5704911	Inf	1.6115806	0.9565946
Cephalopod - GastropodBivalve	0.7616249	0.3267785	Inf	2.3307067	0.5672467
Cephalopod - Medusozoa	0.5063651	0.3085793	Inf	1.6409563	0.9497313
Cephalopod - Nematoda	1.5908998	0.3720475	Inf	4.2760668	0.0017888
Cephalopod - Nudibranch	0.9710629	0.2890740	Inf	3.3592191	0.0552852
Cephalopod - Osteichthyes	-0.3587415	0.3320545	Inf	-1.0803695	0.9991443
Cephalopod - Pinneped	1.5469824	0.3124118	Inf	4.9517419	0.0000744
Cephalopod - Polychaete	1.0849459	0.3205293	Inf	3.3848569	0.0510165
Cephalopod - Porifera	1.0618293	0.3462832	Inf	3.0663605	0.1288848
Cephalopod - Testudines	0.8443127	0.3211999	Inf	2.6286209	0.3503222
Cetacea - Anthozoa	-0.5656852	0.4129030	Inf	-1.3700194	0.9898304
Cetacea - Echinoderm	-0.3669098	0.4225381	Inf	-0.8683473	0.9999329
Cetacea - Elasmobranch	-0.7265580	0.6490714	Inf	-1.1193807	0.9987340
Cetacea - GastropodBivalve	-0.8843255	0.4330281	Inf	-2.0421897	0.7729916
Cetacea - Medusozoa	-1.1395852	0.4151944	Inf	-2.7447030	0.2777011
Cetacea - Nematoda	-0.0550505	0.4573552	Inf	-0.1203671	1.0000000
Cetacea - Nudibranch	-0.6748874	0.4035515	Inf	-1.6723698	0.9415561
Cetacea - Osteichthyes	-2.0046918	0.4413829	Inf	-4.5418427	0.0005444
Cetacea - Pinneped	-0.0989679	0.4196454	Inf	-0.2358370	1.0000000
Cetacea - Polychaete	-0.5610044	0.4161140	Inf	-1.3481989	0.9912929
Cetacea - Porifera	-0.5841211	0.4594762	Inf	-1.2712759	0.9951457
Cetacea - Testudines	-0.8016376	0.4332549	Inf	-1.8502679	0.8773552

contrast	estimate	SE	df	z.ratio	p.value
Anthozoa - Echinoderm	0.1987753	0.2781469	Inf	0.7146414	0.9999940
Anthozoa - Elasmobranch	-0.1608729	0.5528095	Inf	-0.2910096	1.0000000
Anthozoa - GastropodBivalve	-0.3186403	0.2941993	Inf	-1.0830764	0.9991201
Anthozoa - Medusozoa	-0.5739000	0.2761923	Inf	-2.0779005	0.7499872
Anthozoa - Nematoda	0.5106346	0.3475017	Inf	1.4694448	0.9804466
Anthozoa - Nudibranch	-0.1092023	0.2556775	Inf	-0.4271094	1.0000000
Anthozoa - Osteichthyes	-1.4390067	0.3070704	Inf	-4.6862430	0.0002758
Anthozoa - Pinneped	0.4667173	0.2791891	Inf	1.6716890	0.9417427
Anthozoa - Polychaete	0.0046808	0.2976144	Inf	0.0157276	1.0000000
Anthozoa - Porifera	-0.0184359	0.3194435	Inf	-0.0577126	1.0000000
Anthozoa - Testudines	-0.2359524	0.2850373	Inf	-0.8277949	0.9999625
Echinoderm - Elasmobranch	-0.3596482	0.5443720	Inf	-0.6606663	0.9999978
Echinoderm - GastropodBivalve	-0.5174156	0.2828683	Inf	-1.8291748	0.8866282
Echinoderm - Medusozoa	-0.7726754	0.2771777	Inf	-2.7876538	0.2532638
Echinoderm - Nematoda	0.3118593	0.3479623	Inf	0.8962446	0.9999017
Echinoderm - Nudibranch	-0.3079776	0.2538193	Inf	-1.2133732	0.9969980
Echinoderm - Osteichthyes	-1.6377820	0.3033462	Inf	-5.3990523	0.0000069
Echinoderm - Pinneped	0.2679419	0.2773955	Inf	0.9659203	0.9997617
Echinoderm - Polychaete	-0.1940946	0.2963662	Inf	-0.6549146	0.9999980
Echinoderm - Porifera	-0.2172112	0.3091662	Inf	-0.7025710	0.9999952
Echinoderm - Testudines	-0.4347278	0.2781293	Inf	-1.5630422	0.9663837
Elasmobranch - GastropodBivalve	-0.1577674	0.5574903	Inf	-0.2829958	1.0000000
Elasmobranch - Medusozoa	-0.4130272	0.5518503	Inf	-0.7484405	0.9999893
Elasmobranch - Nematoda	0.6715075	0.6022259	Inf	1.1150425	0.9987867
Elasmobranch - Nudibranch	0.0516706	0.5404495	Inf	0.0956068	1.0000000
Elasmobranch - Osteichthyes	-1.2781338	0.5635312	Inf	-2.2680799	0.6145727
Elasmobranch - Pinneped	0.6275901	0.5477623	Inf	1.1457344	0.9983693
Elasmobranch - Polychaete	0.1655536	0.5667437	Inf	0.2921137	1.0000000
Elasmobranch - Porifera	0.1424370	0.5659860	Inf	0.2516616	1.0000000
Elasmobranch - Testudines	-0.0750796	0.5475343	Inf	-0.1371230	1.0000000

contrast	estimate	SE	df	z.ratio	p.value
GastropodBivalve - Medusozoa	-0.2552597	0.2897539	Inf	-0.8809534	0.9999201
GastropodBivalve - Nematoda	0.8292749	0.3575761	Inf	2.3191567	0.5760099
GastropodBivalve - Nudibranch	0.2094380	0.2691748	Inf	0.7780746	0.9999826
GastropodBivalve - Osteichthyes	-1.1203664	0.3226524	Inf	-3.4723631	0.0384983
GastropodBivalve - Pinneped	0.7853576	0.2902220	Inf	2.7060580	0.3008459
GastropodBivalve - Polychaete	0.3233211	0.3146926	Inf	1.0274187	0.9995142
GastropodBivalve - Porifera	0.3002044	0.3275688	Inf	0.9164622	0.9998717
GastropodBivalve - Testudines	0.0826879	0.2907214	Inf	0.2844230	1.0000000
Medusozoa - Nematoda	1.0845346	0.3485703	Inf	3.1113799	0.1141604
Medusozoa - Nudibranch	0.4646978	0.2481755	Inf	1.8724561	0.8671127
Medusozoa - Osteichthyes	-0.8651067	0.3128426	Inf	-2.7653101	0.2658046
Medusozoa - Pinneped	1.0406173	0.2680863	Inf	3.8816507	0.0089519
Medusozoa - Polychaete	0.5785808	0.2930922	Inf	1.9740572	0.8139773
Medusozoa - Porifera	0.5554641	0.3179586	Inf	1.7469700	0.9184542
Medusozoa - Testudines	0.3379476	0.2804275	Inf	1.2051159	0.9972054
Nematoda - Nudibranch	-0.6198369	0.3332590	Inf	-1.8599254	0.8729585
Nematoda - Osteichthyes	-1.9496413	0.3738496	Inf	-5.2150421	0.0000189
Nematoda - Pinneped	-0.0439173	0.3540852	Inf	-0.1240305	1.0000000
Nematoda - Polychaete	-0.5059539	0.3638202	Inf	-1.3906701	0.9882682
Nematoda - Porifera	-0.5290705	0.3868265	Inf	-1.3677206	0.9899933
Nematoda - Testudines	-0.7465871	0.3549668	Inf	-2.1032586	0.7330829
Nudibranch - Osteichthyes	-1.3298044	0.2908124	Inf	-4.5727225	0.0004716
Nudibranch - Pinneped	0.5759195	0.2477999	Inf	2.3241318	0.5722362
Nudibranch - Polychaete	0.1138830	0.2768097	Inf	0.4114126	1.0000000
Nudibranch - Porifera	0.0907663	0.2954676	Inf	0.3071956	1.0000000
Nudibranch - Testudines	-0.1267502	0.2580919	Inf	-0.4911049	1.0000000
Osteichthyes - Pinneped	1.9057240	0.3163586	Inf	6.0239367	0.0000002
Osteichthyes - Polychaete	1.4436874	0.3348424	Inf	4.3115426	0.0015334
Osteichthyes - Porifera	1.4205708	0.3378504	Inf	4.2047335	0.0024273
Osteichthyes - Testudines	1.2030542	0.3108073	Inf	3.8707394	0.0093340

contrast	estimate	SE	df	z.ratio	p.value
Pinneped - Polychaete	-0.4620365	0.2938632	Inf	-1.5722843	0.9646620
Pinneped - Porifera	-0.4851532	0.3187246	Inf	-1.5221705	0.9732476
Pinneped - Testudines	-0.7026697	0.2792138	Inf	-2.5166011	0.4281855
Polychaete - Porifera	-0.0231167	0.3450168	Inf	-0.0670016	1.0000000
Polychaete - Testudines	-0.2406332	0.3121226	Inf	-0.7709573	0.9999845
Porifera - Testudines	-0.2175165	0.3168480	Inf	-0.6865012	0.9999964

```
kable(cld(marginal_taxa,
    alpha   = 0.05,
    Letters = letters,    ### Use lower-case letters for .group
    type    = "response", ### Report emmeans in orginal scale
    adjust =  "tukey"))  %>%
  kable_styling(bootstrap_options = c("striped", "hover", "condensed"))
```

	Taxa	response	SE	df	asymp.LCL	asymp.UCL	.group
3	Cetacea	22.27194	8.229733	Inf	7.549436	65.70546	ab
9	Nematoda	23.53240	6.834725	Inf	10.054610	55.07659	ab
12	Pinneped	24.58891	4.841033	Inf	13.816646	43.75984	a
5	Echinoderm	32.14437	6.061637	Inf	18.506584	55.83205	ab
13	Polychaete	39.03005	8.749482	Inf	20.246838	75.23865	abc
4	Anthozoa	39.21317	7.777233	Inf	21.940537	70.08363	ab
14	Porifera	39.94280	9.943137	Inf	19.271336	82.78759	abc
10	Nudibranch	43.73789	7.171984	Inf	27.061847	70.69005	abc
6	Elasmobranch	46.05726	23.596376	Inf	10.276868	206.41225	abcd
15	Testudines	49.64834	10.111052	Inf	27.349797	90.12711	abc
7	GastropodBivalve	53.92817	11.600792	Inf	28.727007	101.23739	abc
1	Arthropod	56.57670	10.522842	Inf	32.820350	97.52862	abc
8	Medusozoa	69.61031	13.650007	Inf	39.204656	123.59743	bcd
2	Cephalopod	115.50084	27.426201	Inf	57.630889	231.48079	cd
11	Osteichthyes	165.34254	39.226883	Inf	82.550509	331.16881	d

```
marginal_photo = emmeans(like_model_negbin, ~ Photo.Type )

kable(pairs(marginal_photo, adjust="tukey"))  %>%
  kable_styling(bootstrap_options = c("striped", "hover", "condensed"))
```

contrast	estimate	SE	df	z.ratio	p.value
Behavioral - Color	-0.3892771	0.1444331	Inf	-2.6952073	0.0192701
Behavioral - Standard Scientific	-0.4529471	0.1438014	Inf	-3.1498096	0.0046546
Color - Standard Scientific	-0.0636700	0.1374774	Inf	-0.4631308	0.8885173

```
kable(cld(marginal_photo,
    alpha   = 0.05,
    Letters = letters,    ### Use lower-case letters for .group
    type    = "response", ### Report emmeans in orginal scale
    adjust =  "tukey")) %>%
  kable_styling(bootstrap_options = c("striped", "hover", "condensed"))
```

Photo.Type	response	SE	df	asymp.LCL	asymp.UCL	.group
Behavioral	35.11736	3.761951	Inf	27.19163	45.35327	a
Color	51.83019	5.385305	Inf	40.44245	66.42447	b
Standard Scientific	55.23754	5.697907	Inf	43.17842	70.66459	b

```
marginal_awe = emmeans(like_model_negbin, ~ Awe.Factor )

kable(pairs(marginal_awe, adjust="tukey")) %>%
  kable_styling(bootstrap_options = c("striped", "hover", "condensed"))
```

contrast	estimate	SE	df	z.ratio	p.value
High - Low	0.6797694	0.1159276	Inf	5.863741	0

```
kable(cld(marginal_awe,
    alpha   = 0.05,
    Letters = letters,    ### Use lower-case letters for .group
    type    = "response", ### Report emmeans in orginal scale
    adjust =  "tukey")) %>%
  kable_styling(bootstrap_options = c("striped", "hover", "condensed"))
```

	Awe.Factor	response	SE	df	asymp.LCL	asymp.UCL	.group
2	Low	33.10063	2.935946	Inf	27.14471	40.36336	a
1	High	65.32154	5.604981	Inf	53.91553	79.14052	b

Reduced Model

```r
#examine a reduced model
  drop1(like_model_negbin, test="F")
```

```
## Single term deletions
##
## Model:
## Like.24 ~ Caption.Type + Taxa + Photo.Type + Awe.Factor + Days +
##     Time + LogCapCount
##              Df Deviance    AIC F value    Pr(>F)
## <none>             76.759 723.75
## Caption.Type  1   77.650 722.64  0.6271   0.43189
## Taxa         14  159.679 778.67  4.1668 6.902e-05 ***
## Photo.Type    2   86.796 729.79  3.5306   0.03622 *
## Awe.Factor    1  108.444 753.43 22.2911 1.705e-05 ***
## Days          1   77.880 722.87  0.7892   0.37829
## Time          1   76.762 721.75  0.0028   0.95824
## LogCapCount   1   76.823 721.81  0.0452   0.83252
## ---
## Signif. codes:  0 '***' 0.001 '**' 0.01 '*' 0.05 '.' 0.1 ' ' 1
```

```r
  like_model_negbin2 = glm.nb(data=fb_data, Like.24~
                              Taxa+
                              Photo.Type+
                              Awe.Factor,
                            control = glm.control(maxit=10000))
  #anova
  Anova(like_model_negbin2,
        type="II",
        test="LR")
```

```
## Analysis of Deviance Table (Type II tests)
##
## Response: Like.24
##            LR Chisq Df Pr(>Chisq)
## Taxa         89.366 14  4.999e-13 ***
## Photo.Type   12.366  2   0.002064 **
## Awe.Factor   30.458  1  3.412e-08 ***
## ---
## Signif. codes:  0 '***' 0.001 '**' 0.01 '*' 0.05 '.' 0.1 ' ' 1
```

```r
  #pseudo r squared
  nagelkerke(like_model_negbin2)
```

```
## $Models
##

## Model: "glm.nb, Like.24 ~ Taxa + Photo.Type + Awe.Factor, fb_data, glm.control(maxit
= 10000), 4.754659395, log"
## Null:  "glm.nb, Like.24 ~ 1, fb_data, glm.control(maxit = 10000), 1.535934784, log"

##
## $Pseudo.R.squared.for.model.vs.null
##                              Pseudo.R.squared
## McFadden                             0.117719
## Cox and Snell (ML)                   0.698208
## Nagelkerke (Cragg and Uhler)         0.698235
##
## $Likelihood.ratio.test
##  Df.diff LogLik.diff  Chisq    p.value
##      -17     -45.525 91.049 3.9319e-12
##
## $Number.of.observations
##
## Model: 76
## Null:  76
##
## $Messages
## [1] "Note: For models fit with REML, these statistics are based on refitting with ML"
##
## $Warnings
## [1] "None"
```

```
AIC(like_model_negbin, like_model_negbin2)
```

```
##                    df      AIC
## like_model_negbin  23 725.7492
## like_model_negbin2 19 720.3962
```

Predicting New Data

```
newdata1 <- data.frame(Taxa = "Cephalopod", Photo.Type="Color", Awe.Factor="High")
temp <- predict(like_model_negbin2, newdata1, type = "response",se.fit = TRUE)
newdata1$predicted <- temp$fit
newdata1$se <-temp$se.fit
newdata1$upr <- with(newdata1, predicted + (2 *se))
newdata1$lwr <- with(newdata1, predicted - (2 *se))
newdata1
```

```
##         Taxa Photo.Type Awe.Factor predicted       se      upr      lwr
## 1 Cephalopod      Color       High  192.1423 49.22081 290.5839 93.70071
```

Predicting Shares Analyses

```
hist(fb_data$Share.24,breaks=50)
```

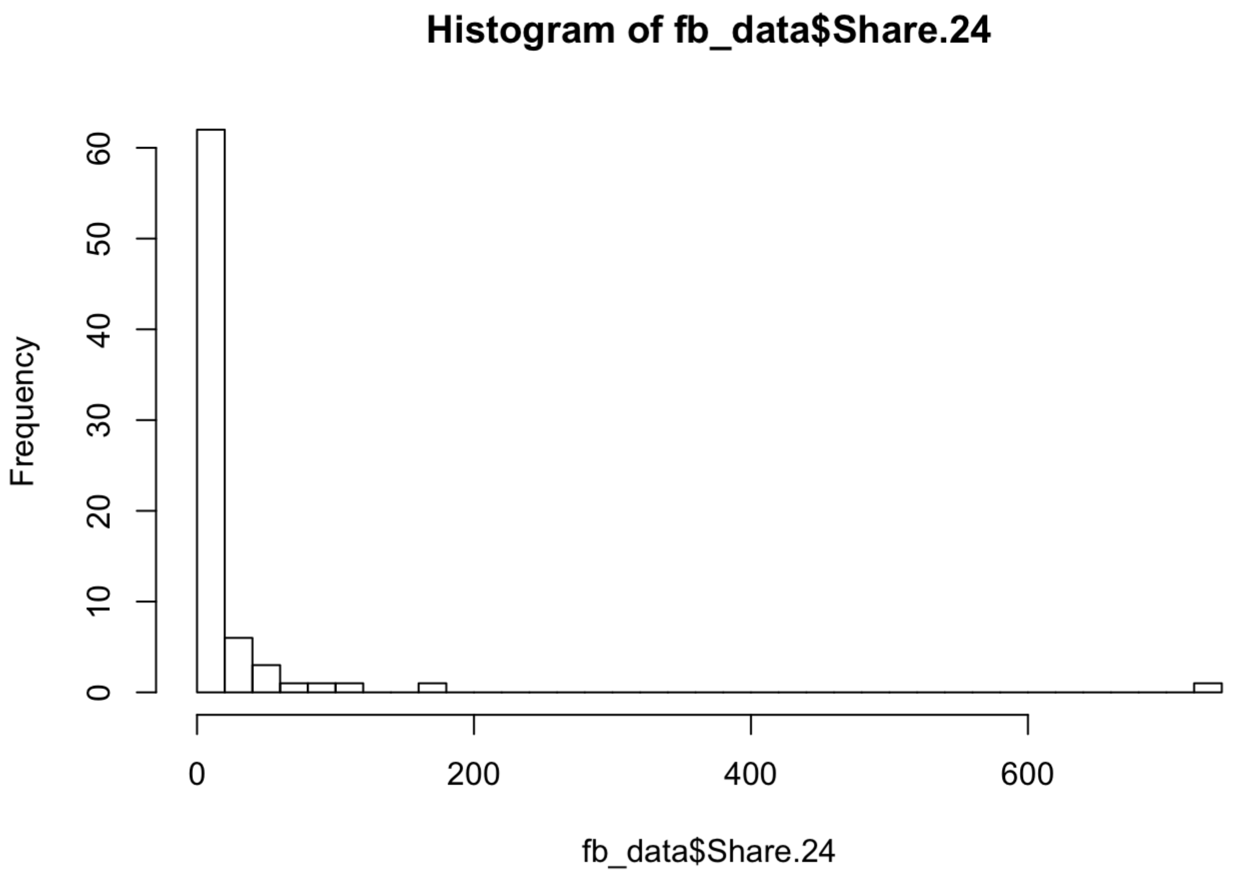


```
summary(fb_data$Share.24)
```

```
##    Min. 1st Qu.  Median    Mean 3rd Qu.    Max.
##    0.00    1.00    2.00   22.55   14.00  734.00
```

```
sum(fb_data$Share.24 == 0)
```

```
## [1] 16
```

Large number of zeros as well so will need a zero inflation model, Obivously not a Poisson model but will run to show overdispersion

```
share_model_poisson<-glm(data=fb_data, Share.24~
                          Caption.Type+
                          Taxa+
                          Photo.Type+
                          Awe.Factor+
                          Days+
                          Time+
                          LogCapCount, family=poisson)
summary(share_model_poisson)
```

```
##
## Call:
## glm(formula = Share.24 ~ Caption.Type + Taxa + Photo.Type + Awe.Factor +
##     Days + Time + LogCapCount, family = poisson, data = fb_data)
##
## Deviance Residuals:
##    Min      1Q  Median      3Q     Max
## -5.974  -2.045  -0.305   1.387   9.951
##
## Coefficients:
##                                 Estimate Std. Error z value Pr(>|z|)
## (Intercept)                    0.6867524  0.4577767   1.500   0.1336
## Caption.TypeScientific        -0.0420110  0.1099183  -0.382   0.7023
## TaxaCephalopod                 1.3609375  0.1660297   8.197 2.47e-16 ***
## TaxaCetacea                   -4.4916874  1.0125417  -4.436 9.16e-06 ***
## TaxaAnthozoa                  -2.1238852  0.3622176  -5.864 4.53e-09 ***
## TaxaEchinoderm                -2.1134751  0.3853431  -5.485 4.14e-08 ***
## TaxaElasmobranch               0.4902708  1.0156700   0.483   0.6293
## TaxaGastropodBivalve          -0.4588181  0.2349693  -1.953   0.0509 .
## TaxaMedusozoa                  1.2317491  0.1628754   7.563 3.95e-14 ***
## TaxaNematoda                  -1.2091789  0.2523730  -4.791 1.66e-06 ***
## TaxaNudibranch                -0.9401280  0.1993747  -4.715 2.41e-06 ***
## TaxaOsteichthyes               3.2812872  0.1722161  19.053  < 2e-16 ***
## TaxaPinneped                  -1.0579549  0.2589029  -4.086 4.38e-05 ***
## TaxaPolychaete                -0.3077627  0.1811469  -1.699   0.0893 .
## TaxaPorifera                  -1.3463437  0.2972084  -4.530 5.90e-06 ***
## TaxaTestudines                 0.4594421  0.2099533   2.188   0.0286 *
## Photo.TypeColor                1.7557973  0.1190380  14.750  < 2e-16 ***
## Photo.TypeStandard Scientific  0.9536351  0.1393788   6.842 7.81e-12 ***
## Awe.FactorLow                 -2.0843036  0.0830566 -25.095  < 2e-16 ***
## Days                           0.0026976  0.0002846   9.477  < 2e-16 ***
## Time                           2.0385961  0.2742650   7.433 1.06e-13 ***
## LogCapCount                   -0.5151964  0.2351599  -2.191   0.0285 *
## ---
## Signif. codes:  0 '***' 0.001 '**' 0.01 '*' 0.05 '.' 0.1 ' ' 1
##
## (Dispersion parameter for poisson family taken to be 1)
##
##     Null deviance: 6384.13  on 75  degrees of freedom
## Residual deviance:  646.92  on 54  degrees of freedom
## AIC: 909.92
##
## Number of Fisher Scoring iterations: 7
```

```
#need to test for overdispersion
dispersiontest(share_model_poisson,trafo=1)
```

```
##
##  Overdispersion test
##
## data:  share_model_poisson
## z = 2.5267, p-value = 0.005757
## alternative hypothesis: true alpha is greater than 0
## sample estimates:
##    alpha
## 13.98815
```

Model is over-dispersed with p-value=0.005757. Proceeding with Negative binomical regerssion

```
share_model_negbin = glm.nb(data=fb_data, Share.24~
                              Caption.Type+
                              Taxa+
                              Photo.Type+
                              Awe.Factor+
                              Days+
                              Time+
                              LogCapCount,
                            control = glm.control(maxit=10000))
  #dispersion test
  odTest(share_model_negbin)
```

```
## Likelihood ratio test of H0: Poisson, as restricted NB model:
## n.b., the distribution of the test-statistic under H0 is non-standard
## e.g., see help(odTest) for details/references
##
## Critical value of test statistic at the alpha= 0.05 level: 2.7055
## Chi-Square Test Statistic =  424.4769 p-value = < 2.2e-16
```

Because Zero-inflation runs two models on zeros and non-zeros particularly sensitive to number of states in independent variables. Working to reduced taxa down to post hoc groups of social media performance.

```
fb_data <- fb_data %>%
  mutate(Taxa2 = dplyr::recode(Taxa,
           Cephalopod = "Charismatic",
           Osteichthyes = "Charismatic",
           Medusozoa = "Charismatic",
           Arthropod = "Average",
           Anthozoa = "Average",
           Porifera = "Average",
           Nudibranch = "Average",
           Nematoda = "Average",
           Testudines = "Average",
           Polychaete = "Average",
           GastropodBivalve = "Low",
           Elasmobranch = "Low",
           Cetacea = "Low",
           Pinneped = "Low",
           Echinoderm = "Low"))
        summary(fb_data$Taxa2)
```

```
##     Average Charismatic         Low
##          40          14          22
```

```
        share_model_zi3 <- zeroinfl(data=fb_data, Share.24~
                                       Photo.Type+
                                       Awe.Factor+
                                       Taxa2+
                                      Caption.Type,
                                     dist = "negbin")

         summary(share_model_zi3)
```

```
##
## Call:
## zeroinfl(formula = Share.24 ~ Photo.Type + Awe.Factor + Taxa2 +
##     Caption.Type, data = fb_data, dist = "negbin")
##
## Pearson residuals:
##     Min      1Q  Median      3Q     Max
## -0.8202 -0.6276 -0.3475  0.1259  6.7564
##
## Count model coefficients (negbin with log link):
##                               Estimate Std. Error z value Pr(>|z|)
## (Intercept)                     2.3172     0.4021   5.762 8.29e-09 ***
## Photo.TypeColor                 0.5434     0.4296   1.265  0.20590
## Photo.TypeStandard Scientific   0.9012     0.4512   1.997  0.04580 *
## Awe.FactorLow                  -1.1329     0.3517  -3.221  0.00128 **
## Taxa2Charismatic                1.9962     0.4272   4.673 2.97e-06 ***
## Taxa2Low                       -1.1040     0.4017  -2.749  0.00599 **
## Caption.TypeScientific         -0.7118     0.3461  -2.057  0.03971 *
## Log(theta)                     -0.3692     0.1947  -1.896  0.05796 .
##
## Zero-inflation model coefficients (binomial with logit link):
##                               Estimate Std. Error z value Pr(>|z|)
## (Intercept)                     -3.995      3.627  -1.101    0.271
## Photo.TypeColor                  1.033      2.123   0.487    0.626
## Photo.TypeStandard Scientific  -13.617    944.220  -0.014    0.988
## Awe.FactorLow                    2.964      3.492   0.849    0.396
## Taxa2Charismatic               -14.377   1914.399  -0.008    0.994
## Taxa2Low                        -8.827    259.298  -0.034    0.973
## Caption.TypeScientific         -15.261   1845.447  -0.008    0.993
## ---
## Signif. codes:  0 '***' 0.001 '**' 0.01 '*' 0.05 '.' 0.1 ' ' 1
##
## Theta = 0.6913
## Number of iterations in BFGS optimization: 63
## Log-likelihood: -228.5 on 15 Df
```

```
        #anova
        Anova(share_model_zi3,
              type="II",
              test="Chisq")
```

```
## Analysis of Deviance Table (Type II tests)
##
## Response: Share.24
##              Df   Chisq Pr(>Chisq)
## Photo.Type    2  4.0076   0.134825
## Awe.Factor    1 10.3761   0.001277 **
## Taxa2         2 45.5624  1.277e-10 ***
## Caption.Type  1  4.2302   0.039711 *
## ---
## Signif. codes:  0 '***' 0.001 '**' 0.01 '*' 0.05 '.' 0.1 ' ' 1
```

```
#pseudo r squared
nagelkerke(share_model_zi3)
```

```
## $Models
##

## Model: "zeroinfl, Share.24 ~ Photo.Type + Awe.Factor + Taxa2 + Caption.Type, fb_data,
negbin"
## Null:  "zeroinfl, Share.24 ~ 1, fb_data, negbin"

##
## $Pseudo.R.squared.for.model.vs.null
##                              Pseudo.R.squared
## McFadden                             0.130343
## Cox and Snell (ML)                   0.593996
## Nagelkerke (Cragg and Uhler)         0.594586
##
## $Likelihood.ratio.test
##  Df.diff LogLik.diff  Chisq    p.value
##      -12     -34.253 68.506 6.0901e-10
##
## $Number.of.observations
##
## Model: 76
## Null:  76
##
## $Messages
## [1] "Note: For models fit with REML, these statistics are based on refitting with ML"
##
## $Warnings
## [1] "None"
```

Comparisons

```
#comparisons
marginal_taxa_share = emmeans(share_model_zi3, ~ Taxa2 )

kable(pairs(marginal_taxa_share, adjust="tukey")) %>%
  kable_styling(bootstrap_options = c("striped", "hover", "condensed"))
```

contrast	estimate	SE	df	z.ratio	p.value
Average - Charismatic	-55.352553	21.801831	Inf	-2.538895	0.0299131
Average - Low	5.382469	2.234562	Inf	2.408735	0.0423193
Charismatic - Low	60.735022	21.651618	Inf	2.805103	0.0139348

```
kable(cld(marginal_taxa_share,
    alpha   = 0.05,
    Letters = letters,    ### Use lower-case letters for .group
    type    = "response", ### Report emmeans in orginal scale
    adjust =  "tukey")) %>%
  kable_styling(bootstrap_options = c("striped", "hover", "condensed"))
```

	Taxa2	emmean	SE	df	asymp.LCL	asymp.UCL	.group
3	Low	2.864393	0.9154111	Inf	0.6786308	5.050154	a
1	Average	8.246862	2.0744133	Inf	3.2937064	13.200017	b
2	Charismatic	63.599415	21.6474831	Inf	11.9108992	115.287931	c

```
marginal_caption_share = emmeans(share_model_zi3, ~ Caption.Type )
kable(pairs(marginal_caption_share, adjust="tukey")) %>%
  kable_styling(bootstrap_options = c("striped", "hover", "condensed"))
```

contrast	estimate	SE	df	z.ratio	p.value
Public - Scientific	16.84228	9.831124	Inf	1.713159	0.0866833

```
kable(cld(marginal_caption_share,
    alpha   = 0.05,
    Letters = letters,    ### Use lower-case letters for .group
    type    = "response", ### Report emmeans in orginal scale
    adjust =  "tukey")) %>%
  kable_styling(bootstrap_options = c("striped", "hover", "condensed"))
```

	Caption.Type	emmean	SE	df	asymp.LCL	asymp.UCL	.group
2	Scientific	16.48242	5.502529	Inf	4.176140	28.78869	a
1	Public	33.32470	11.089264	Inf	8.523816	58.12557	a

```
marginal_awe_share = emmeans(share_model_zi3, ~ Awe.Factor )
kable(pairs(marginal_awe_share, adjust="tukey"))%>%
  kable_styling(bootstrap_options = c("striped", "hover", "condensed"))
```

contrast	estimate	SE	df	z.ratio	p.value
High - Low	25.81829	10.47777	Inf	2.464102	0.0137357

```
kable(cld(marginal_awe_share ,
    alpha   = 0.05,
    Letters = letters,    ### Use lower-case letters for .group
    type    = "response", ### Report emmeans in orginal scale
    adjust =  "tukey")) %>%
  kable_styling(bootstrap_options = c("striped", "hover", "condensed"))
```

	Awe.Factor	emmean	SE	df	asymp.LCL	asymp.UCL	.group
2	Low	11.99441	4.384578	Inf	2.188405	21.80042	a
1	High	37.81270	11.857124	Inf	11.294521	64.33088	b

```
marginal_photo_share = emmeans(share_model_zi3, ~ Photo.Type )

kable(pairs(marginal_photo_share, adjust="tukey")) %>%
  kable_styling(bootstrap_options = c("striped", "hover", "condensed"))
```

contrast	estimate	SE	df	z.ratio	p.value
Behavioral - Color	-10.23653	8.028569	Inf	-1.2750127	0.4093210
Behavioral - Standard Scientific	-21.27547	11.109246	Inf	-1.9151140	0.1343717
Color - Standard Scientific	-11.03895	12.398015	Inf	-0.8903801	0.6463878

```
kable(cld(marginal_photo_share ,
    alpha   = 0.05,
    Letters = letters,    ### Use lower-case letters for .group
    type    = "response", ### Report emmeans in orginal scale
    adjust =  "tukey"))%>%
  kable_styling(bootstrap_options = c("striped", "hover", "condensed"))
```

Photo.Type	emmean	SE	df	asymp.LCL	asymp.UCL	.group
Behavioral	14.39956	6.797455	Inf	-1.830986	30.63010	a
Color	24.63608	8.860603	Inf	3.479288	45.79288	a
Standard Scientific	35.67503	12.105033	Inf	6.771384	64.57867	a

Predicting Shares Plots

```
p1s <- ggplot(fb_data, aes(Awe.Factor,LogShares))+
    geom_boxplot(fill="#5B9A9B", color="black", alpha=.8, notch=FALSE)+
    theme_bw(base_size=10)+
    theme(panel.grid.major = element_line(size = .2, color = "grey"))+
    xlab("Awe Factor")+
    ylab("Log10 Facebook Shares")+
    theme(axis.text.x = element_text(angle = 45, hjust = 1))

  p2s <- ggplot(fb_data, aes(Caption.Type, LogShares))+
    geom_boxplot(fill="#5B9A9B", color="black", alpha=.8, notch=FALSE)+
    theme_bw(base_size=10)+
    theme(panel.grid.major = element_line(size = .2, color = "grey"))+
    xlab("Caption Type")+
    ylab("Log10 Facebook Shares")+
    theme(axis.text.x = element_text(angle = 45, hjust = 1))

  p4s <- ggplot(fb_data, aes(Taxa2, LogLikes))+
    geom_boxplot(fill="#5B9A9B", color="black", alpha=.8, notch=FALSE)+
    theme_bw(base_size=10)+
    theme(panel.grid.major = element_line(size = .2, color = "grey"))+
    xlab("Taxon Group")+
    ylab("Log10 Facebook Shares")+
    theme(axis.text.x = element_text(angle = 45, hjust = 1))+
    scale_x_discrete(labels = wrap_format(15))

  grid.arrange(p1s,p2s,p4s, ncol=1)
```

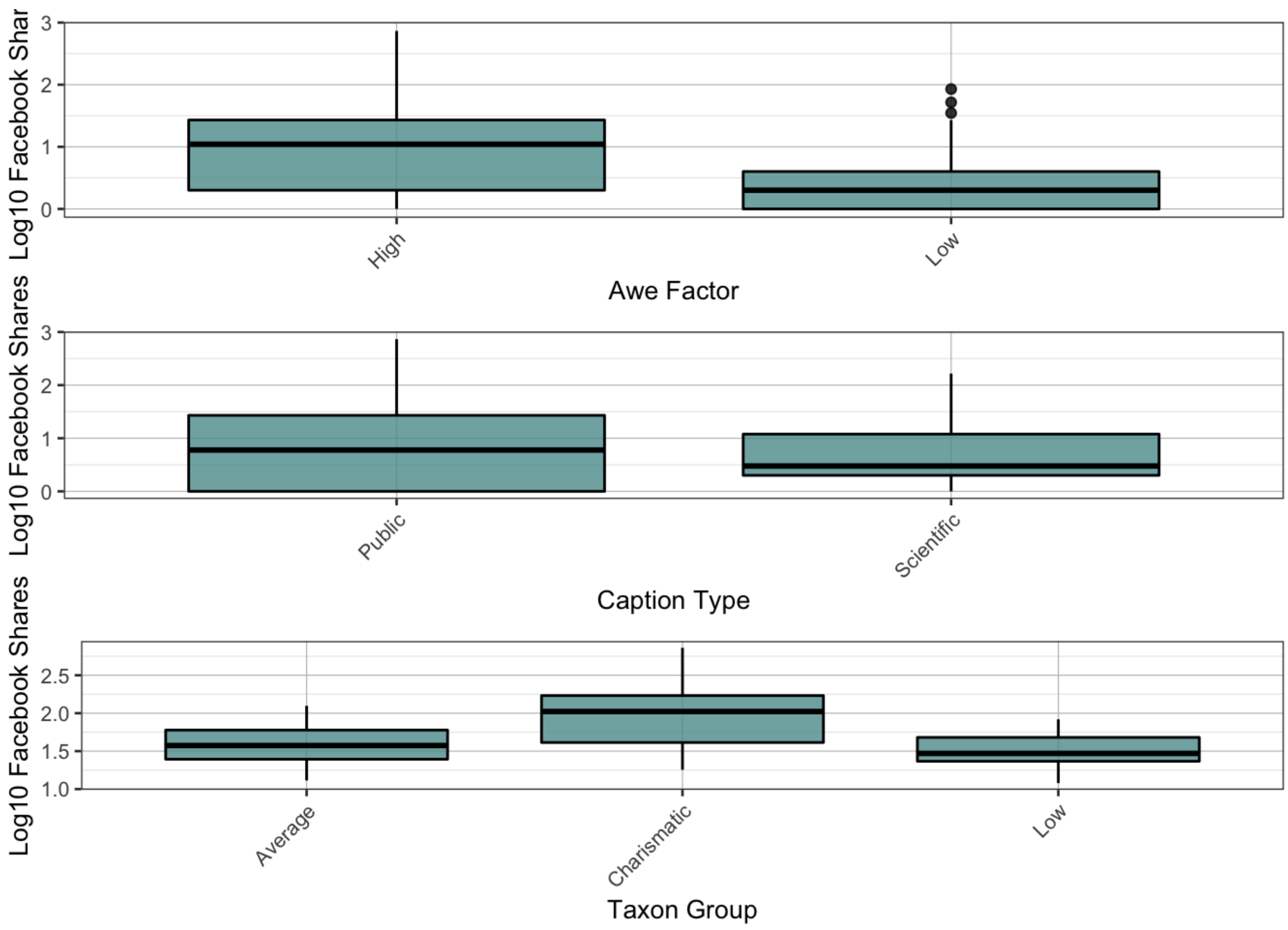

```
pdf(file="figure4.pdf",width=8.5, height=11, useDingbats=FALSE)
par(mar=c(5,3,2,2)+0.1) #removes space from around edges of pdf
 grid.arrange(p1s,p2s,p4s, ncol=1)
```

```
## Warning: Removed 16 rows containing non-finite values (stat_boxplot).

## Warning: Removed 16 rows containing non-finite values (stat_boxplot).
```

```
dev.off()
```

```
## quartz_off_screen
##                 2
```

Predicting Comments

```
hist(fb_data$Comment.24,breaks=50)
```

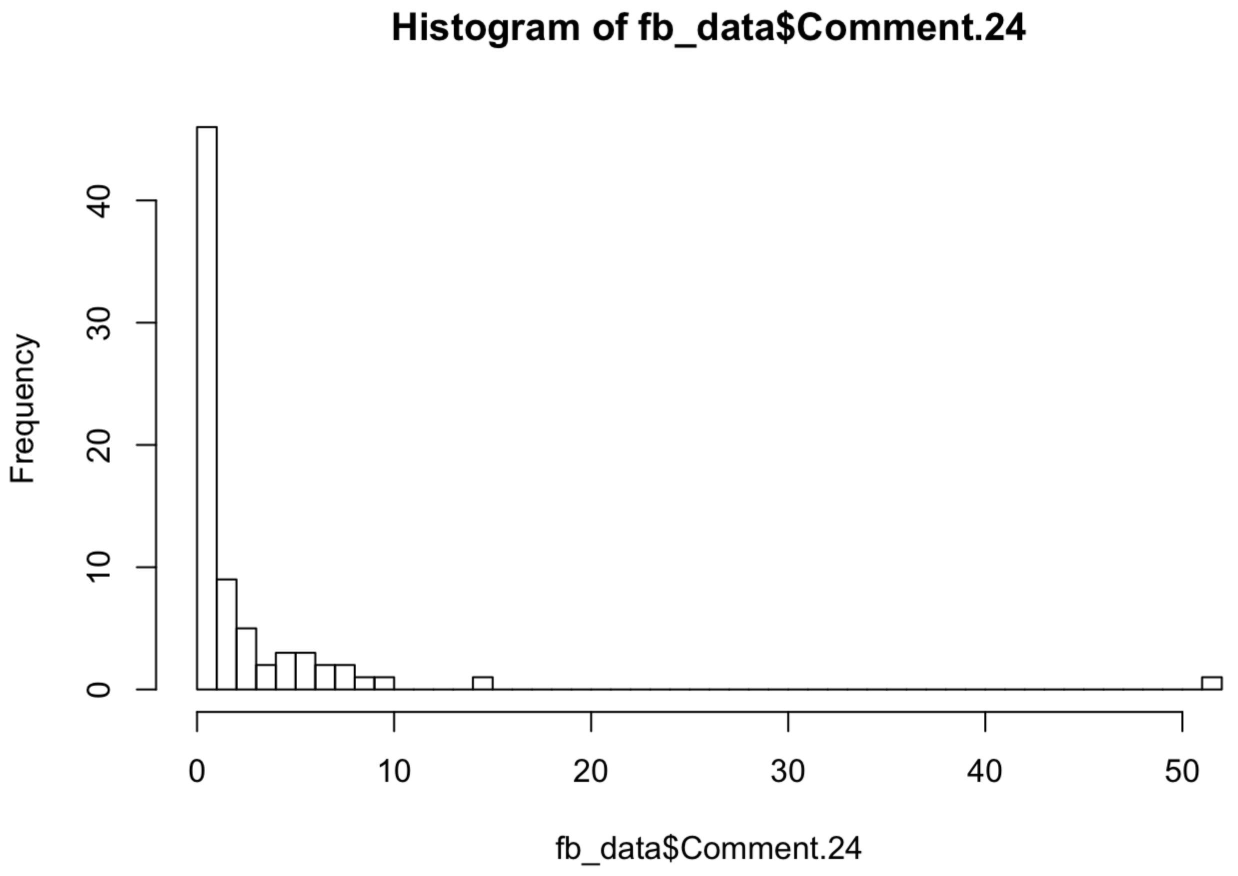


```
        summary(fb_data$Comment.24)
```

```
##    Min. 1st Qu.  Median    Mean 3rd Qu.    Max. 
##   0.000   0.000   1.000   2.737   3.000  52.000
```

```
sum(fb_data$Comment.24 == 0)
```

```
## [1] 28
```

Heavily zero inflated so will need a zero infaltion model. Although obviously inccored, Will run Poisson model to demonstrate overdisperson.

```
comment_model_poisson<-glm(data=fb_data, Comment.24~
                           Caption.Type+
                           Taxa+
                           Photo.Type+
                           Awe.Factor+
                           Days+
                           Time+
                           LogCapCount, family=poisson)
summary(comment_model_poisson)
```

```
##
## Call:
## glm(formula = Comment.24 ~ Caption.Type + Taxa + Photo.Type +
##     Awe.Factor + Days + Time + LogCapCount, family = poisson,
##     data = fb_data)
##
## Deviance Residuals:
##     Min       1Q   Median       3Q      Max
## -3.4117  -1.4435  -0.3396   0.4354   4.3699
##
## Coefficients:
##                                Estimate Std. Error z value Pr(>|z|)
## (Intercept)                  -3.867e-01  9.809e-01  -0.394   0.6934
## Caption.TypeScientific       -9.808e-02  2.540e-01  -0.386   0.6994
## TaxaCephalopod                2.958e-01  3.723e-01   0.794   0.4270
## TaxaCetacea                  -9.836e-01  5.416e-01  -1.816   0.0694 .
## TaxaAnthozoa                 -1.058e+00  4.722e-01  -2.240   0.0251 *
## TaxaEchinoderm               -1.062e+00  5.330e-01  -1.992   0.0464 *
## TaxaElasmobranch             -1.477e+01  1.276e+03  -0.012   0.9908
## TaxaGastropodBivalve          7.166e-01  3.715e-01   1.929   0.0537 .
## TaxaMedusozoa                -4.394e-01  4.221e-01  -1.041   0.2979
## TaxaNematoda                  1.706e-01  3.924e-01   0.435   0.6638
## TaxaNudibranch               -5.329e-01  3.976e-01  -1.340   0.1801
## TaxaOsteichthyes              1.512e+00  3.469e-01   4.359 1.31e-05 ***
## TaxaPinneped                 -9.850e-01  5.090e-01  -1.935   0.0530 .
## TaxaPolychaete               -4.048e-01  3.661e-01  -1.106   0.2688
## TaxaPorifera                 -2.311e+00  1.068e+00  -2.163   0.0305 *
## TaxaTestudines                2.931e-01  4.428e-01   0.662   0.5080
## Photo.TypeColor               3.661e-01  2.398e-01   1.526   0.1269
## Photo.TypeStandard Scientific  4.705e-01  2.449e-01   1.921   0.0547 .
## Awe.FactorLow                -1.062e+00  1.690e-01  -6.287 3.25e-10 ***
## Days                          3.041e-03  5.535e-04   5.494 3.92e-08 ***
## Time                          1.244e+00  6.661e-01   1.868   0.0618 .
## LogCapCount                  -5.899e-02  4.917e-01  -0.120   0.9045
## ---
## Signif. codes:  0 '***' 0.001 '**' 0.01 '*' 0.05 '.' 0.1 ' ' 1
##
## (Dispersion parameter for poisson family taken to be 1)
##
##     Null deviance: 472.85  on 75  degrees of freedom
## Residual deviance: 184.57  on 54  degrees of freedom
## AIC: 365.05
##
## Number of Fisher Scoring iterations: 13
```

```
#need to test for overdispersion
dispersiontest(comment_model_poisson,trafo=1)
```

```
##
##  Overdispersion test
##
## data:  comment_model_poisson
## z = 2.5527, p-value = 0.005344
## alternative hypothesis: true alpha is greater than 0
## sample estimates:
##    alpha
## 1.650175
```

Model is over dispersed with p-value = 0.005344. Proceeding with zero-inflation Negative binomial regression

```
comment_model_zi3 <- zeroinfl(data=fb_data, Comment.24~
                                Photo.Type+
                                Awe.Factor+
                                Caption.Type+
                                Taxa2,
                              dist = "negbin")


#anova
summary(comment_model_zi3)
```

```
##
## Call:
## zeroinfl(formula = Comment.24 ~ Photo.Type + Awe.Factor + Caption.Type +
##     Taxa2, data = fb_data, dist = "negbin")
##
## Pearson residuals:
##     Min      1Q  Median      3Q     Max
## -0.9609 -0.6873 -0.3333  0.2466  2.9687
##
## Count model coefficients (negbin with log link):
##                               Estimate Std. Error z value Pr(>|z|)
## (Intercept)                     1.0471     0.3634   2.881 0.003963 **
## Photo.TypeColor                 0.4599     0.3884   1.184 0.236280
## Photo.TypeStandard Scientific   1.2607     0.4177   3.018 0.002543 **
## Awe.FactorLow                  -1.1363     0.3168  -3.587 0.000335 ***
## Caption.TypeScientific         -0.2717     0.3334  -0.815 0.414975
## Taxa2Charismatic                0.4255     0.4158   1.023 0.306138
## Taxa2Low                       -0.6047     0.3502  -1.727 0.084215 .
## Log(theta)                      0.1583     0.3153   0.502 0.615722
##
## Zero-inflation model coefficients (binomial with logit link):
##                                Estimate Std. Error z value Pr(>|z|)
## (Intercept)                    -11.6775   205.3860  -0.057    0.955
## Photo.TypeColor                 10.0708   205.3845   0.049    0.961
## Photo.TypeStandard Scientific   11.1765   205.3864   0.054    0.957
## Awe.FactorLow                   -2.3202     1.5642  -1.483    0.138
## Caption.TypeScientific           1.7738     1.1919   1.488    0.137
## Taxa2Charismatic                -0.4147     1.2687  -0.327    0.744
## Taxa2Low                       -20.0879  8578.6030  -0.002    0.998
## ---
## Signif. codes:  0 '***' 0.001 '**' 0.01 '*' 0.05 '.' 0.1 ' ' 1
##
## Theta = 1.1715
## Number of iterations in BFGS optimization: 32
## Log-likelihood: -144.2 on 15 Df
```

```
Anova(comment_model_zi3,
      type="II",
      test="Chisq")
```

```
## Analysis of Deviance Table (Type II tests)
##
## Response: Comment.24
##              Df   Chisq Pr(>Chisq)
## Photo.Type    2  9.4010  0.0090908 **
## Awe.Factor    1 12.8660  0.0003346 ***
## Caption.Type  1  0.6645  0.4149745
## Taxa2         2  6.2720  0.0434561 *
## ---
## Signif. codes:  0 '***' 0.001 '**' 0.01 '*' 0.05 '.' 0.1 ' ' 1
```

```
#pseudo r squared
nagelkerke(comment_model_zi3)
```

```
## $Models
##
## Model: "zeroinfl, Comment.24 ~ Photo.Type + Awe.Factor + Caption.Type + Taxa2, fb_dat
a, negbin"
## Null:  "zeroinfl, Comment.24 ~ 1, fb_data, negbin"
##
## $Pseudo.R.squared.for.model.vs.null
##                              Pseudo.R.squared
## McFadden                            0.0902428
## Cox and Snell (ML)                  0.3136770
## Nagelkerke (Cragg and Uhler)        0.3185940
##
## $Likelihood.ratio.test
##  Df.diff LogLik.diff  Chisq  p.value
##      -12     -14.303 28.607 0.004505
##
## $Number.of.observations
##
## Model: 76
## Null:  76
##
## $Messages
## [1] "Note: For models fit with REML, these statistics are based on refitting with ML"
##
## $Warnings
## [1] "None"
```

Comparisons

```
#comparisons
marginal_taxa_share = emmeans(comment_model_zi3, ~ Taxa2 )

kable(pairs(marginal_taxa_share, adjust="tukey")) %>%
  kable_styling(bootstrap_options = c("striped", "hover", "condensed"))
```

contrast	estimate	SE	df	z.ratio	p.value
Average - Charismatic	-1.505469	1.4490136	Inf	-1.0389612	0.5522318
Average - Low	0.419793	0.7340568	Inf	0.5718808	0.8351143
Charismatic - Low	1.925262	1.4452570	Inf	1.3321243	0.3772768

```
kable(cld(marginal_taxa_share,
    alpha   = 0.05,
    Letters = letters,    ### Use lower-case letters for .group
    type    = "response", ### Report emmeans in orginal scale
    adjust =  "tukey")) %>%
  kable_styling(bootstrap_options = c("striped", "hover", "condensed"))
```

	Taxa2	emmean	SE	df	asymp.LCL	asymp.UCL	.group
3	Low	1.845130	0.5383217	Inf	0.5597589	3.130501	a
1	Average	2.264923	0.5866835	Inf	0.8640767	3.665770	a
2	Charismatic	3.770392	1.3275810	Inf	0.6004766	6.940307	a

```
marginal_awe_share = emmeans(comment_model_zi3, ~ Awe.Factor )

kable(pairs(marginal_awe_share, adjust="tukey")) %>%
  kable_styling(bootstrap_options = c("striped", "hover", "condensed"))
```

contrast	estimate	SE	df	z.ratio	p.value
High - Low	2.109299	0.9662963	Inf	2.18287	0.0290454

```
kable(cld(marginal_awe_share ,
    alpha   = 0.05,
    Letters = letters,    ### Use lower-case letters for .group
    type    = "response", ### Report emmeans in orginal scale
    adjust =  "tukey")) %>%
  kable_styling(bootstrap_options = c("striped", "hover", "condensed"))
```

	Awe.Factor	emmean	SE	df	asymp.LCL	asymp.UCL	.group
2	Low	1.572165	0.3926847	Inf	0.6939353	2.450396	a
1	High	3.681465	0.9272555	Inf	1.6076793	5.755250	b

```
marginal_photo_share = emmeans(comment_model_zi3, ~ Photo.Type )

kable(pairs(marginal_photo_share, adjust="tukey")) %>%
  kable_styling(bootstrap_options = c("striped", "hover", "condensed"))
```

contrast	estimate	SE	df	z.ratio	p.value
Behavioral - Color	-0.4909891	0.7666506	Inf	-0.640434	0.7977694
Behavioral - Standard Scientific	-2.2883253	1.3191388	Inf	-1.734712	0.1922568
Color - Standard Scientific	-1.7973362	1.3496550	Inf	-1.331700	0.3775100

```
kable (cld(marginal_photo_share ,
    alpha   = 0.05,
    Letters = letters,    ### Use lower-case letters for .group
    type    = "response", ### Report emmeans in orginal scale
    adjust =  "tukey")) %>%
  kable_styling(bootstrap_options = c("striped", "hover", "condensed"))
```

Photo.Type	emmean	SE	df	asymp.LCL	asymp.UCL	.group
Behavioral	1.700377	0.5304073	Inf	0.4339034	2.966850	a
Color	2.191366	0.6408684	Inf	0.6611402	3.721592	a
Standard Scientific	3.988702	1.2295589	Inf	1.0528377	6.924567	a

Predicting Comments Plots

```
####plots
p1c <- ggplot(fb_data, aes(Awe.Factor,LogComments))+
  geom_boxplot(fill="#BDBC89", color="black", alpha=.8, notch=FALSE)+
  theme_bw(base_size=10)+
  theme(panel.grid.major = element_line(size = .2, color = "grey"))+
  xlab("Awe Factor")+
  ylab("Log10 Facebook Comments")+
  theme(axis.text.x = element_text(angle = 45, hjust = 1))

p2c <- ggplot(fb_data, aes(Photo.Type, LogComments))+
  geom_boxplot(fill="#BDBC89", color="black", alpha=.8, notch=FALSE)+
  theme_bw(base_size=10)+
  theme(panel.grid.major = element_line(size = .2, color = "grey"))+
  xlab("Photo Type")+
  ylab("Log10 Facebook Comments")+
  theme(axis.text.x = element_text(angle = 45, hjust = 1))

p4c <- ggplot(fb_data, aes(Taxa2, LogComments))+
  geom_boxplot(fill="#BDBC89", color="black", alpha=.8, notch=FALSE)+
  theme_bw(base_size=10)+
  theme(panel.grid.major = element_line(size = .2, color = "grey"))+
  xlab("Taxon Group")+
  ylab("Log10 Facebook Comments")+
  theme(axis.text.x = element_text(angle = 45, hjust = 1))+
  scale_x_discrete(labels = wrap_format(15))

grid.arrange(p1c,p2c,p4c, ncol=1)
```

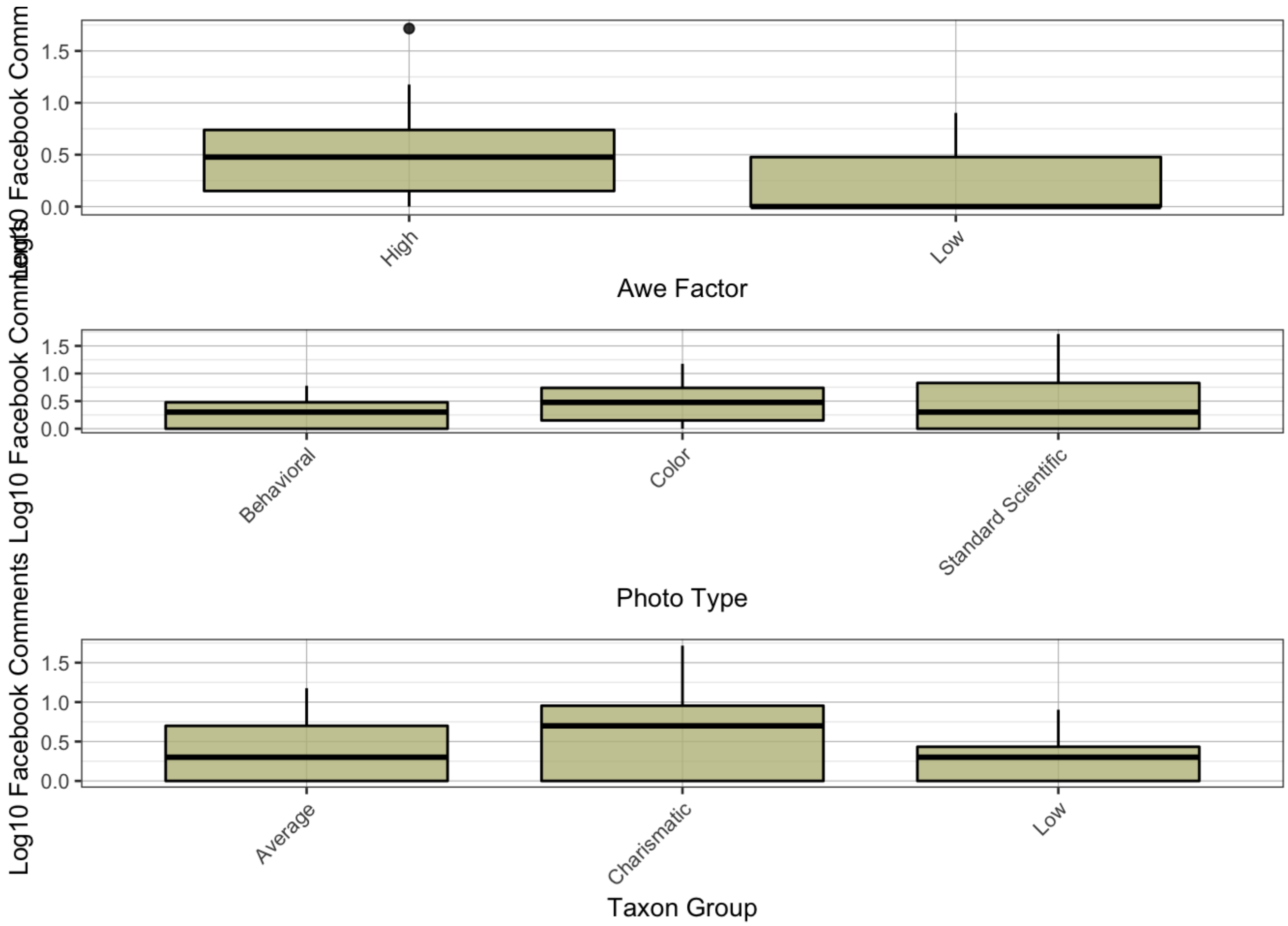


```
pdf(file="figure5.pdf",width=8.5, height=11, useDingbats=FALSE)
par(mar=c(5,3,2,2)+0.1) #removes space from around edges of pdf
 grid.arrange(p1c,p2c,p4c, ncol=1)
```

```
## Warning: Removed 28 rows containing non-finite values (stat_boxplot).

## Warning: Removed 28 rows containing non-finite values (stat_boxplot).

## Warning: Removed 28 rows containing non-finite values (stat_boxplot).
```

```
dev.off()
```

```
## quartz_off_screen
##                 2
```

Favorite Taxa Summary Data

```
setwd("~/Desktop/Facebook Paper/Facebook Experiment")
fav_taxa <- read.csv("favorite_taxa(DSN).csv")
kable(summary(fav_taxa$Taxa)) %>%
    kable_styling(bootstrap_options = c("striped", "hover", "condensed"))
```

	x
	2
Annelida	3
Arthropoda	10
Bivalve	1
Cephalopoda	16
Cetacea	9
Cnidaria	5
Echinodermata	4
Elasmobranchii	10
Mammalia	1
Nudibranchia	6
Osteichthyes	22
Porifiera	1
Testudines	1
